# Intertwined Topological Phases in TaAs_2_ Nanowires with Giant Magnetoresistance and Quantum Coherent Surface Transport

**DOI:** 10.1002/adma.202418279

**Published:** 2025-03-27

**Authors:** Anand Roy, Anna Eyal, Roni Majlin Skiff, Barun Barick, Samuel D. Escribano, Olga Brontvein, Katya Rechav, Ora Bitton, Roni Ilan, Ernesto Joselevich

**Affiliations:** ^1^ Department of Molecular Chemistry and Materials Science Weizmann Institute of Science Rehovot 76100 Israel; ^2^ Physics Department Technion Haifa 32000 Israel; ^3^ Raymond and Beverly Sackler School of Physics and Astronomy Tel Aviv 69978 Israel; ^4^ Department of Condensed Matter Physics Weizmann Institute of Science Rehovot 76100 Israel; ^5^ Chemical Research Support Weizmann Institute of Science Rehovot 76100 Israel

**Keywords:** Aharonov–Bohm oscillations, Dirac cones, giant magnetoresistance, nanowires, topological crystalline insulator, topological semimetal, weak topological insulator

## Abstract

Nanowires (NWs) of topological materials are emerging as an exciting platform to probe and engineer new quantum phenomena that are hard to access in bulk phase. Their quasi‐1D geometry and large surface‐to‐bulk ratio unlock new expressions of topology and highlight surface states. TaAs_2_, a compensated semimetal, is a topologically rich material harboring nodal‐line, weak topological insulator (WTI), C_2_‐protected topological crystalline insulator, and Zeeman field‐induced Weyl semimetal phases. We report the synthesis of TaAs_2_ NWs in situ encapsulated in a dielectric SiO_2_ shell, which enable to probe rich magnetotransport phenomena, including metal‐to‐insulator transition and strong signatures of topologically nontrivial transport at remarkably high temperatures, direction‐dependent giant positive, and negative magnetoresistance, and a double pattern of Aharonov–Bohm oscillations, demonstrating coherent surface transport consistent with the two Dirac cones of a WTI surface. The SiO_2_‐encapsulated TaAs_2_ NWs show room‐temperature conductivity up to 15 times higher than bulk TaAs_2_. The coexistence and susceptibility of topological phases to external stimuli have potential applications in spintronics and nanoscale quantum technology.

## Graphical abstract

Synthesis of topological semimetal TaAs_2_ nanowires in situ encapsulated with a thin SiO_2_ shell unravel a richness of intertwined topological phases manifested by their magnetotransport features: A near‐room‐temperature metal‐to‐insulator transition, strong expressions of topologically nontrivial surface transport, giant magnetoresistance with direction‐dependent sign reversal, chiral anomaly, and a unique double pattern of Aharonov–Bohm oscillations.

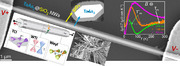

## 1 Introduction

The discovery of new materials with topologically protected electronic bands has been shown to create vast opportunities for fundamental science and technology advances.^[^ ^1^, ^2^ ^]^ In topological semimetals (TSMs), electronic band crossings in the Brillouin zone (BZ) near the Fermi energy (*E*_f_) give rise to bulk Dirac/Weyl/nodal‐line gapless states and distinct topological surface states (TSS).^[^ ^3^ ^]^ These bulk and surface states are expected to have exotic properties potentially stabilized by different crystal symmetries.^[^ ^3^ ^]^ Examining and uncovering the interplay between crystal symmetries and band topologies is thus desirable.

In this aspect, the family of transition metal mono‐ and dipnictides (TX and TX_2_, respectively; wherein T = V, Nb, Ta, La, and X = P, As, Sb) provides a rich platform.^[^ ^4^ ^]^ Noncentrosymmetric TX compounds inherit singly degenerate linear band crossings due to the broken inversion symmetry, and possess Weyl cones of opposite chirality in the bulk and Fermi arcs at the surface.^[^ ^4^, ^5^ ^]^ In contrast, centrosymmetric TX_2_ compounds inheriting doubly degenerate bands due to preserved inversion symmetry, offer bulk nodal‐line states without spin–orbit coupling (SOC), and a rare quantum state of matter, namely 3D weak‐topological‐insulator (WTI), when SOC is included.^[^ ^6^ ^]^ The WTI nature of TaAs_2_ and other TX_2_ analogs is predicted based on its topological invariants (0;111).^[^ ^6^, ^7^ ^]^ Notably, TSS harboring a pair of Dirac cones appear only on specific crystallographic surfaces in WTIs, unlike strong topological insulators (STIs), where all the surfaces must harbor an odd number of Dirac cones.^[^ ^8^, ^9^ ^]^ This anisotropic nature of WTIs enables surface engineering to obtain topologically trivial (gapped) and nontrivial (gapless) surfaces using different orientations of the same crystal.^[^ ^8^ ^]^ Due to this anisotropy, WTIs are rare and have so far been reported in only a few 1D and 2D van der Waals (vdW) materials, including β‐Bi_4_X_4_ (X = Br, I), ZrTe_5_, and Bi_2_TeI.^[^ ^10^, ^11^, ^12^, ^13^, ^14^, ^15^ ^]^ To the best of our knowledge, TX_2_ compounds are the only documented example of inherent 3D WTIs crystals.^[^ ^6^ ^]^ The richness of band topologies in TaAs_2_ and other TX_2_ analogs further stems from the existence of a twofold rotation C_2_ (010) symmetry‐protected topological crystalline insulator (TCI) state with a pair of type‐II Dirac cones on the {010} surfaces.^[^ ^16^ ^]^ In these rotational symmetry‐protected TCIs, Dirac cones associated to the rotation axis can appear not only at high symmetry points but also at generic **k** points in the surface BZ.^[^ ^17^, ^18^ ^]^ These quantum states can further host helical modes on hinges, which could provide a unique platform for realizing and manipulating 0D Majorana zero modes (MZM) in the proximity of a superconductor.^[^ ^17^, ^19^ ^]^ Such rich electronic band topologies in TaAs_2_ and other TX_2_ analogs are expected to bring intriguing properties.

In 2016, three seminal articles reported a magnetic field‐induced metal‐to‐insulator (MI) phase transition, followed by a resistivity plateau, nonsaturating giant magnetoresistance (GMR ≈10^5^ order) with Shubnikov–de Hass (SdH) oscillations, and nontrivial Berry phase in bulk single‐crystals of TaAs_2_.^[^ ^20^, ^21^, ^22^ ^]^ The same year, large negative magnetoresistance (MR) in bulk TaAs_2_ was reported, and its possible origin was linked to the presence of TSS coexisting with a bulk semimetallic state.^[^ ^23^ ^]^ Further, the first angle‐resolved photoemission spectroscopy (ARPES) confirmed the presence of mixed trivial and TSS in TaAs_2_; however, the surface states were poorly visible, and the authors attributed it to the nonperfect surface cleavage.^[^ ^24^ ^]^

It is noteworthy that TSS nurture the potential for high‐speed dissipationless electronics, spintronics, memory devices, and robust quantum bits (qubits).^[^ ^1^, ^2^ ^]^ Nevertheless, identifying and extracting transport of quasiparticles associated with these TSS requires meticulous efforts to suppress overwhelming bulk electronic contribution.^[^ ^2^, ^25^ ^]^ Low‐dimensional systems, e.g., nanowires (NWs), nanoribbons (NRs), thin films, and vdW materials, with pronounced surface‐to‐bulk ratio, provide an essential platform to examine potential surface states and their contribution to transport.^[^ ^1^, ^2^ ^]^ Among different low‐dimensional systems, the NW 1D geometry is particularly interesting owing to its ability to manifest two important phenomena: 1) Aharonov–Bohm (AB) oscillations: oscillations in the electrical resistivity in the presence of a longitudinal magnetic field, which has been observed in NWs (NRs) of TCIs (TIs), attributed to topologically protected metallic surface states,^[^ ^26^ ^]^ and 2) Majorana zero modes (MZM) emerging at interfaces between different topological states along the wires, or as end states, in the proximity of a superconductor.^[^ ^27^ ^]^ MZMs have been proposed as an attractive building block for realizing robust quantum computers.^[^ ^27^, ^28^, ^29^ ^]^ Despite the anticipated rich electronic band topology and transport features in TaAs_2_ and other analogs, an understanding of their surface states and related transport features is absent due to the unavailability of nanostructures from these materials. Thus, it is highly desirable to produce and study low‐dimensional structures of this family of materials.

Besides the lack of nanostructures made of specific topological materials, like TaAs_2_ in this case, a more general problem of topological nanomaterials is that many of them, especially pnictides and chalcogenides, are prone to oxidation, which chemically degrades their surface, and makes surface transport phenomena poorly visible.^[^ ^2^ ^]^ It is thus important to devise a general methodology for producing nanostructures of topological materials wherein the surface is chemically protected.

In this article, we report the synthesis, structure, and magnetotransport properties of topological semimetal TaAs_2_ NWs. These NWs are grown by a chemical vapor‐assisted mechanism that in situ encapsulates them in a thin shell of SiO_2_, which conveniently acts as a protective layer and dielectric for electrical gating. The SiO_2_ shell can be locally etched to make ohmic contacts, while the rest of the NW remains encapsulated. The morphology and single‐crystalline structure of the NWs are examined using scanning electron microscopy (SEM), atomic resolution scanning transmission electron microscopy (STEM) with energy dispersive spectra (EDS), atomic‐resolution chemical mapping, and Raman spectroscopy. NWs with varying TaAs_2_ core diameters are integrated into four‐probe devices to study their rich magnetotransport properties. The NWs exhibit a metallic conductivity that turns into an insulating state in an external magnetic field, resulting in a tunable MI transition. Notably, in the NWs, field‐induced MI transition occurs at nearly four times higher temperatures than their bulk counterpart, reaching close to room temperature. Moreover, a low‐temperature universal resistivity plateau known in the bulk phase is replaced by a previously unobserved nontrivial metallic conduction mode. Another effect of the magnetic field is a highly anisotropic GMR, and its tunability with TaAs_2_ diameter. Finally, the MR sign‐reversal and quantum oscillations in a field perfectly aligned with the NW‐axis are discussed. A theoretical model is proposed to qualitatively explain how these phenomena in the NWs can arise from the different topological phases and the quasi‐1D geometry.

## 2 Results and Discussion

### 2.1 Synthesis and Structural Analysis

**Figure 1:**
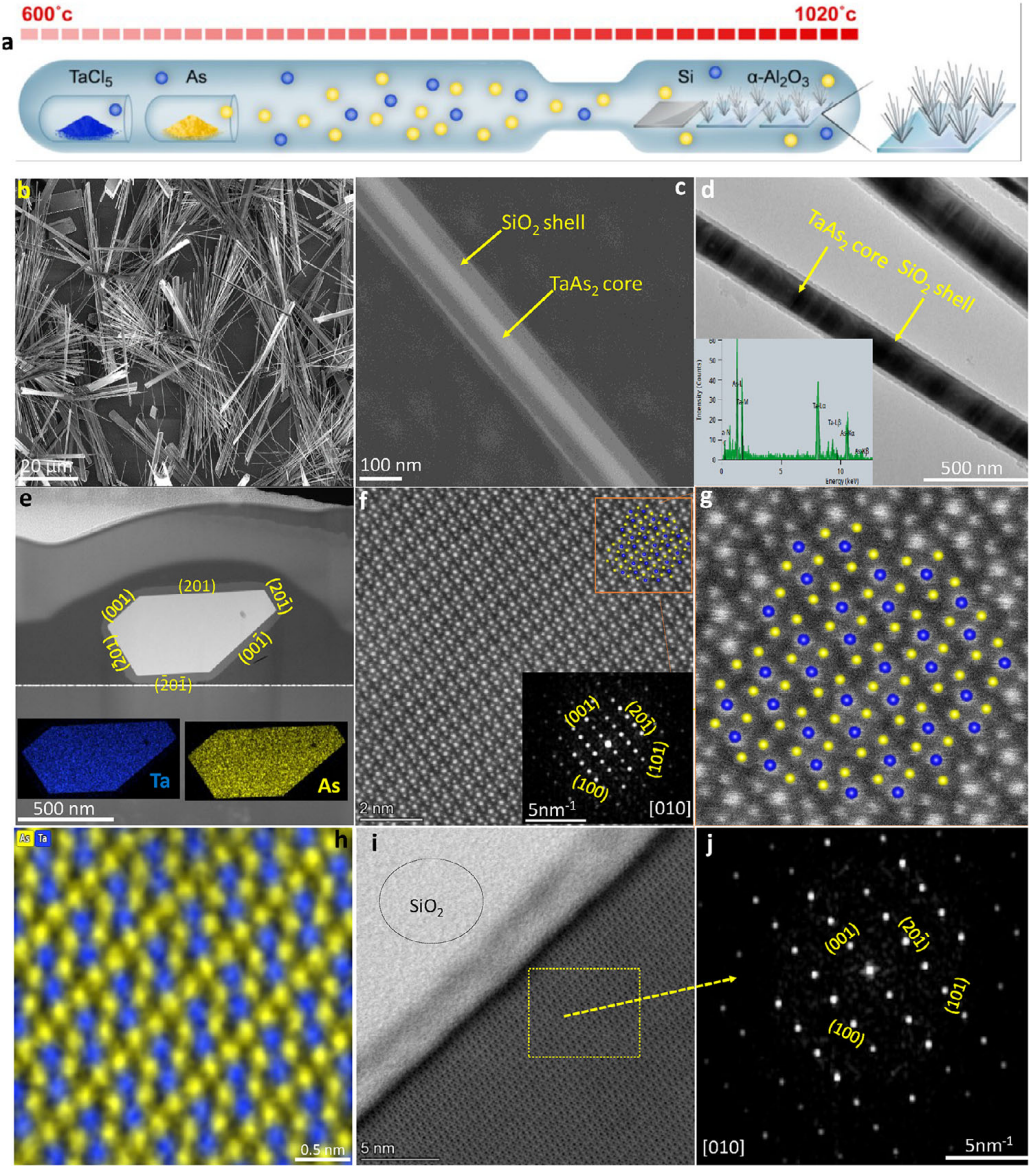
Synthesis and structural analysis of TaAs_2_ NWs. a) Schematic representation of the synthesis apparatus and process. b) Low‐magnification SEM image showing large area growth of NWs. c) High‐magnification SEM image showing a single NW with core‐shell TaAs_2_@SiO_2_ structure. d) Top‐view TEM image of a NWs showing core–shell structure; inset shows EDS spectrum from core with Ta and As peaks. e) Top panel: HAADF‐STEM image showing a cross‐section of NW with TaAs_2_ core, and exposed facets encapsulated in an amorphous SiO_2_ shell. Bottom panels: EDS elemental mapping from the core region. f) Atomic‐resolution HAADF‐STEM image from the NW core showing highly ordered TaAs_2_ lattice; inset shows corresponding FFT pattern and lattice planes of single‐crystalline core. g) Atomic‐resolution fit of NW lattice with reported structure. h) Atomic‐resolution EDS mapping of core showing lattice arrangements of Ta (blue) and As (yellow). i) High‐resolution bright‐field (BF) STEM image of interface showing highly crystalline TaAs_2_ surface with amorphous SiO_2_ shell. j) FFT pattern from interface region showing lattice planes of single‐crystalline TaAs_2_.

We synthesized TaAs_2_ NWs inside a vacuum‐sealed quartz ampule using a chemical vapor reaction of TaCl_5,_ and As precursors in a temperature gradient maintained by physical isolation between the growth (1020 °C) and precursor activation (600 °C) zones using silicon and sapphire substrates (**Figure** **1**a). Low‐magnification SEM images show a large‐area growth of NWs on different plane‐sapphire (C, R, and AM‐α‐Al_2_O_3_) substrates (Figure 1b; and Figures S1 and S2, Supporting Information). The lengths of NWs range from a few tens to a few hundred micrometers, and it can be noticed that some wide nanobelt (NBs) structures also form alongside thin NWs. High‐resolution SEM and TEM reveal that the NWs possess a core–shell structure wherein the single‐crystalline core is uniformly encapsulated by an amorphous SiO_2_ shell (Figure 1c,d). The EDS spectra measured from the ensemble of NWs and from an individual NW core yielded the As:Ta ratio 2.09 (Figure S3 (Supporting Information); and Figure 1d, inset), thus indicating TaAs_2_ phase of the NWs. A survey of SEM and TEM on several samples showed that TaAs_2_ core diameters (*d*) range between 14 and 450 nm, and the SiO_2_ shell around them is 60–80 nm thick. A plausible pathway for the SiO_2_ shell formation is the generation of SiCl_4_ due to the reactivity of Si substrate with in situ released Cl_2_ vapor, and its conversion to SiO_2_ at small partial pressures of O_2_ (please refer to the Experimental Section and Supporting Information for detailed discussion on the synthetic conditions and possible growth mechanism).

To study the crystal structure and orientation, atomic‐resolution STEM was performed on NW cross‐sections prepared by a focused‐ion‐beam (FIB). The upper panel in Figure 1e presents the high angle annular dark field (HAADF) STEM image of a NW cross‐section, showing an irregular hexagonal TaAs_2_ core with a {010} plane cross‐section sided by parallel pairs of {001}, {201}, and {20$\bar{1}$} facets, surrounded by an amorphous SiO_2_ shell.

The EDS elemental mapping of the core and the corresponding spectrum is shown in the lower panel of Figure 1e; and in Figure S4 (Supporting Information), respectively. This high‐resolutions elemental characterization of the nanowire (NW) core confirms the ubiquitous presence of As and Ta, and an As: Ta atomic ratio of 1.94, indicating that the NWs do not have significant cation or anion vacancies compared to the reported bulk TaAs_2_.^[^ ^21^ ^]^ Atomic resolution HAADF STEM image of the core (Figure 1f) and corresponding fast Fourier transformation (FFT) pattern (inset, Figure 1f) reveals the highly ordered single‐crystalline nature of the NW, with (001), (100), (20$\bar{1}$), (101) lattice planes along a zone axis of [010]. The identified planes and corresponding lattice spacing agree with the reported monoclinic crystal structure of TaAs_2_ (C2/m).^[^ ^21^ ^]^ Note that the NW growth direction <010> is the easy crystallization direction of monoclinic TaAs_2_. Figure 1g shows the atomic‐scale fitting between the observed and simulated crystal structure.^[^ ^30^ ^]^ Atomic‐resolution EDS mapping of TaAs_2_ core (Figure 1h) display lattice arrangements of Ta (blue) and As (yellow) atoms wherein each Ta atom surrounded with six As atoms can be seen. It can be noticed that As atoms adopt two different lattice arrangements directed by their different chemical states (As^3−^ and As_2_^4−^). To the best of our knowledge, this is not only the first report of TaAs_2_ NWs but also the first atomic resolution TEM of a TaAs_2_ crystal, which shows the lattice arrangements of Ta and As atoms in the crystal (Figure S5, Supporting Information).

Next, we examined the interface between the single‐crystalline TaAs_2_ core and the amorphous SiO_2_ shell. Atomic resolution STEM image shows a clean interface (Figure 1i) with the FFT pattern under a zone‐axis [010] manifesting (001), (200), (20$\bar{1}$), (101) planes corresponding to single‐crystalline TaAs_2_ surface with no signs of alloying or substitution or any impurity (Figure 1j). In Figures S6 and S7 (Supporting Information), we show cross‐sectional STEM and crystal structure analysis from a different NW confirming its TaAs_2_ phase. Furthermore, a single‐NW‐based Raman spectrum recorded from multiple NWs synthesized at growth temperature ranging from 915 to 1035 °C and TaCl_5_:As molar ratio ranging from 1:1 to 1:4 show a pure phase of TaAs_2_ with the characteristic peaks that agree with the reported TaAs_2_ spectrum (Figure S8, Supporting Information).^[^ ^30^ ^]^

### 2.2 Electrical and Magnetotransport Properties in a Transversal Magnetic Field

**Figure 2:**
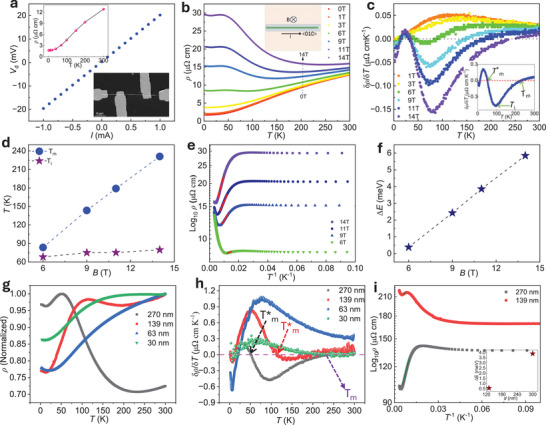
Transport properties of TaAs_2_ NWs in a perpendicular magnetic field configuration. a) A four‐probe current–voltage (*I*–*V*) curve from a TaAs_2_ NW *d* ≈ 300 nm; lower inset show SEM image of the device; upper inset show resistivity versus temperature (RT) in the absence of magnetic field. b) RT curves of the same device under increasing field intensities (dotted black arrow represents increasing 0–14 T). Inset shows schematic representation of top‐view NW with applied electric and magnetic field directions. c) Corresponding first‐derivative of resistivity δ*ρ*/δ*T* as a function of temperature; Inset show metal‐to‐insulator (MI) and insulator‐to‐metal (IM) transition as a function of temperature. d) MI transition temperature (*T*_m_) and inflection point temperature (*T*_i_) obtained from (c) as a function of field intensity. e) Arrhenius log_10_ *ρ* versus *T* ^−1^ plots of the insulating phase under increasing field strengths for the same device. f) Obtained insulating gaps from (e) as a function of magnetic field strength. g) *RT* curves for NWs with varying TaAs_2_ core *d* ≈ 270, 139, and 63, and 30 nm at 14 T field. h) Corresponding δ*ρ*/δ*T* versus temperature plots showing MI (*T*_m_) and IM (T^*^_m_) transitions with respect to NWs diameter. i) Arrhenius log_10_ *ρ* versus *T* ^−1^ plot for NWs (*d* ≈ 270 and 139 nm); inset shows recorded insulating gap in these NWs at 14 T.

For studying the magnetotransport properties, individual NWs, as shown in Figure 1, were integrated into four‐terminal devices (Figure S9, Supporting Information). Contacts were made by local etching of the SiO_2_ shell immediately followed by metal deposition (see the Experimental Section). The electrical conductivity of NWs with TaAs_2_ core diameter (*d*) ranging from 30–450 nm exhibited Ohmic contacts, and ampacity (current carrying capacity) as high as 3–4 mA under ambient conditions (Figure S10, Supporting Information). **Figure** **2**a shows a typical room‐temperature *I*–*V* characteristic curve from a NW (Figure 2a lower‐inset SEM image) with *d* ≈ 300 nm. The electrical behavior of the NWs exhibits a metallic nature, with their resistivity starting to saturate near 25 K (Figure 2a, upper inset). Notably, all NWs with *d* range ≈14–450 nm exhibit a metallic conductivity with their low temperature (2 K) resistivity as low as 1.7–35 µΩ cm.

Remarkably, the room temperature resistivity of these TaAs_2_ NWs ranges from 0.012 to 0.334 mΩ cm, which is up to 15 times smaller than the values reported for the bulk‐single‐crystals TaAs_2_ (0.2–0.8 mΩ cm) (Figure S11, Supporting Information).^[^ ^21^, ^22^, ^23^, ^24^ ^]^ Generally, topologically trivial materials in their nanostructure forms show much reduced conductivity than their bulk counterpart due to increased surface scattering.^[^ ^31^, ^32^ ^]^ It is expected from topologically nontrivial materials that their topologically protected states (TSS) would provide protection against backscattering and other surface perturbations, thus giving rise to the uncompromised conductivity in the nanostructures with respect to their bulk counterpart.^[^ ^2^, ^33^ ^]^ However, to the best of our knowledge, it is rare to obtain such a phenomenon even in topological materials, e.g., topological insulators (TIs) and semimetals (TSMs), due to charge doping of bulk electronic states (in TIs)^[^ ^34^ ^]^ or overwhelming bulk semimetallic band transport (TSMs).^[^ ^35^ ^]^ One example wherein nanostructures shows superior conductivity than their bulk counterpart was recently reported in relatively thicker nanobelts of Weyl semimetal NbAs.^[^ ^36^ ^]^ In our TaAs_2_ NWs, even the thinnest (*d* ≈ 30 nm) NW shows a room temperature resistivity (0.180 mΩ cm) that is lower than the lowest reported value for the bulk. This enhanced conductivity in a reduced dimension, even at room temperature, highlights the power of topological materials at the nanoscale, and their potential use as superior circuit interconnects.^[^ ^37^ ^]^

Application of a magnetic field perpendicular to the NW axis <010> and the current direction, increases the resistivity with a pronounced effect in low‐temperature regions, where a transition from metallic‐to‐insulating (MI) state occurs (Figure 2b). To better analyze the MI transition, the first derivative of the resistivity δ*ρ*/δ*T* with respect to temperature under varying field strengths is shown in Figure 2c. The δ*ρ*/δ*T* changes sign from positive to negative due to the MI transition, and the corresponding transition temperature (*T*_m_) shows a linear increase with the field strength (Figure 2d). It is noteworthy that the MI transition in NWs occurs at much higher temperatures (*T*_m_) than in bulk TaAs_2_.^[^ ^20^, ^21^ ^]^

Notably, at 14 T, *T*_m_ reaches 236 K for a *d* ≈ 300 nm NW device, which is almost 3.5 times higher than the transition temperature (≈60–70 K) reported in bulk TaAs_2_.^[^ ^22^ ^]^ It is technologically appealing that metallic TaAs_2_ NW devices can be tuned into an insulating state near room temperature.

The insulating phase under a magnetic field originates from the formation of cyclotron orbits of carriers along and across the NWs, thus producing quantized energy levels, i.e., Landau levels.^[^ ^38^ ^]^ Insulating gaps under varying fields at *T* ≤ *T*_m_ can be obtained using the Arrhenius equation *ln* ρ (*T*) = ln *K* +  Δ/*K*_B_ *T* (Figure 2e; and Figure S12, Supporting Information). The linear region fit of a ln *ρ* versus *T* ^−1^ provides slopes equivalent to Δ/*k*_B_, where Δ *= ℏω*_c –_ *Γ* = *ℏ* (*eB*/*m* ^*^)—*Γ* is the insulating gap linked to the Landau energy levels, *ω*_c_ = *eB*/*m* ^*^ is the cyclotron frequency, *e* is the elementary charge, *m* ^*^ is the effective mass, *B* is field strength, *Γ* is a localization parameter associated to the boundary of NWs, *k*_B_ is Boltzmann constant, and *K* is a constant value. The insulating gaps (Δ in meV) show a linear dependence and increase with the field strength as expected from the above equation (Figure 2f). Note that localization effects are stronger in NWs than their bulk‐counterparts hence, *Γ* is expected to be larger in NWs.^[^ ^39^ ^]^ Therefore, an insulating phase in NWs can only occur, if *ℏ*(*eB*/*m* ^*^) > *Γ*, and the resulting gap will always be smaller than bulk counterparts. This explains the smaller gap (5.8 meV) observed in NWs (*d* ≈ 300 nm) with respect to bulk TaAs_2_ (15.3 meV).^[^ ^22^ ^]^

Notice that with a further decrease in temperature, δ*ρ*/δ*T* reached its minimum, followed by a swift change at the inflection point (*T*_i_) (Figure 2c, inset). In bulk TX_2_ and other semimetals (LaSb, MoAs_2_, WTe_2_), *T*_i_ was used as a marker of the origin of the resistivity plateau.^[^ ^40^, ^41^, ^42^ ^]^ In TaAs_2_ NWs, we observe replacement of the resistivity plateau by a metallic conduction mode manifested by a second change in δ*ρ*/δ*T* from negative to positive, thus giving rise to a nontrivial insulator‐to‐metal (IM) transition at *T* ^*^_m_ (Figure 2c and inset). Furthermore, magnetotransport features at 14 T in NWs with varying TaAs_2_ core (*d* ≈ 270, 139, 63, and 30 nm) demonstrate the field‐induced electrical transitions as a function of temperature (Figure 2g). It can be noticed that features change markedly in different NW core *d*, with 270 nm exhibiting MI transition at *T*_m_ ≈ 236 K. The corresponding δ*ρ*/δ*T* manifests magnetic field‐induced MI and IM transitions (Figure 2h). Notably, the replacement of the resistivity plateau by the metallic conduction mode at *T* ≈ *T*_i_ is enhanced by the higher surface‐to‐bulk ratio in NWs, and the corresponding nontrivial IM transition temperatures (*T* ^*^_m_) increase with the core *d* reduction. A shift of T^*^_m_ from 51 to 115 K due to a reduced *d* (from 270 to 139 nm) is noteworthy (Figure 2h). In *d* ≈ 63, and 30 nm NWs, we did not observe any electrical transition, and it maintained a metallic nature even at 14 T.

In TSMs, a universal resistivity plateau has recently been reported under broken time‐reversal‐symmetry (TRS) in an external magnetic field.^[^ ^40^, ^41^, ^42^ ^]^ The resistivity plateau in LaSb was indicated to originate from TSS.^[^ ^40^ ^]^ The authors provided an analogy with a similar plateau in a TI SmB_6_ under a zero field where TRS was preserved. In MoAs_2_, the appearance of a similar field‐induced resistivity plateau saturating the bulk insulating resistivity at low temperatures was also attributed to TSS transport contribution.^[^ ^41^ ^]^ A similar plateau has also been observed in bulk TX_2_ compounds; in TaSb_2,_ it was attributed to the presence of metallic TSS based on the observation of a nontrivial Berry phase.^[^ ^43^ ^]^

We show in quasi‐1D TaAs_2_ NWs that pronounced surface states lead to the replacement of the resistivity plateau by a dominating metallic surface conduction mode, leading instead to the appearance of a nontrivial IM transition. In light of the rich coexisting electronic band topologies and associated TSS, e.g., WTI,^[^ ^6^, ^7^ ^]^ C_2_‐symmetry‐protected TCI,^[^ ^16^ ^]^ and Zeeman field‐induced Weyl points,^[^ ^7^ ^]^ it is likely to observe a strong TSS expression, particularly in NWs which could enable the strong manifestation of these TSS due to high surface versus bulk transport ratio. Since our NW terminates at the {010} surface, the observed TSS transport cannot be associated with the TCI phase, which is predicted to host type‐II Dirac fermions on that surface.^[^ ^16^ ^]^ However, the WTI phase of TaAs_2_ is predicted to have a metallic surface state on the {001}, {201}, and {20$\bar{1}$} surfaces (Figure 1e) that can potentially contribute to the observed surface conduction.^[^ ^6^ ^]^ It is noteworthy that despite being called “weak” TI, the surface states of WTIs actually possess predicted robust features, which brings about richness in the transport.^[^ ^8^ ^]^ Also, the surface Fermi arcs associated with Zeeman field‐induced bulk Weyl cones cannot be ignored as a potential source of surface conduction.^[^ ^7^ ^]^ Note that WTI phase in 1D and 2D vdW layered crystals, e.g., β‐Bi_4_X_4_ (X = Br, and I), ZrTe_5_, and Bi_2_TeI, have been probed spectroscopically by measuring quasi‐1D surface Dirac electronic states on their side surfaces.^[^ ^11^, ^14^, ^15^ ^]^ However, their magnotransport features are still unknown. It is notable that the 3D‐WTI candidate TaAs_2_, in its NW geometry obtained here, exhibits well‐defined facets that could potentially host these unique topologically protected electronic states, which manifest strongly in the measured magnetotransport. It is of great technological significance that quasi‐1D TaAs_2_ NWs maintain TSS transport features at notably higher temperatures (Figure 2h) than known topological semimetals.

Returning to the discussion on the insulating state, the gaps linked to the MI transition in thinner NWs decrease (Figure 2i, inset) due to an increase in localization parameter (*Γ*), which would enforce a stronger magnetic field to obtain the condition *ℏ*(*eB*/*m* ^*^) > *Γ* as discussed above.^[^ ^39^ ^]^ Magnetotransport studies performed on a number of NWs manifested robust transport features (Figures S13 and S14, Supporting Information). Interestingly, upon further cooling, an insulating upturn in resistivity emerges at ultralow temperatures (*T* ≤ 12 K) under a 14 T field (Figure 2h,i), an effect that nearly vanishes in a NW of 30 nm diameter (*d*). This insulating upturn could be due to a Kondo effect at high magnetic fields.^[^ ^44^ ^]^ Charge vacancies within the NWs may induce localized states at low densities and temperatures, acting as effective magnetic moments under strong magnetic fields. This, in turn, can enhance scattering processes and contributes to the insulating behavior.^[^ ^45^ ^]^ A more rigorous analysis of resistivity at low temperatures and high magnetic fields is needed to discern the origin of this phenomenon.

### 2.3 Giant Magnetoresistance (GMR) in a Transversal Magnetic Field

**Figure 3:**
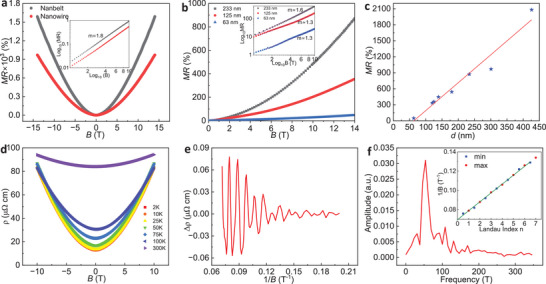
Giant magnetoresistance (GMR) and quantum oscillations in a perpendicular field configuration. a) Field dependent magnetoresistance (MR) in a TaAs_2_ nanowire (nanobelt) of 300 nm thickness (diameter) at 2 K; inset shows log *MR* versus log *B* plot with a quadratic field dependence. b) MR in NWs with varying TaAs_2_ core, *d* ≈ 233, 125, and 63 nm; Inset shows log *MR* versus log *B* plots and obtained slops (m). c) MR as a function of NW core diameter *d* (red solid line represent linear fit). d) Temperature dependence of MR in a NW (*d* ≈ 270 nm). e) Shubnikov–de Haas (SdH) oscillations from the same device at 2 K. f) FFT spectrum of SdH‐oscillations; inset shows Landau fan diagram with blue (red) circles representing Δ *ρ* min. (max.), respectively.

A second consequence of TRS breaking is the occurrence of a field‐dependent GMR. In **Figure** **3**a, we present the MR (% *MR* = (*ρ*_B_−*ρ*_0_)/*ρ*_0_ × 100) measured at 2 K in two independent devices NW (NB) with TaAs_2_ core diameter (thickness) ≈300 nm. A nonsaturating GMR of the order 10^3^% was observed in both devices. The log *MR* versus log *B* plot yielded a linear dependence with a slope of 1.8 ± 0.05, indicating a close to quadratic dependence (inset, Figure 3a). Note that the quadratic dependence of MR in TaAs_2_ NWs is consistent with the similar dependence observed in the bulk TX_2_, associated to the nearly perfect electrons and holes compensation.^[^ ^21^ ^]^

To further understand the behavior of the GMR, we measured MR in devices with varying TaAs_2_ core diameter (*d*) (Figure 3b). In compensated semimetals, MR is derived from the simplification of semiclassical two‐band theory and maintains quadratic dependence even under strong fields as MR *= µ*_e_ *µ*_h_ *B* ^2^, wherein *µ*_e_ and *µ*_h_ are the mobilities of electrons and holes, respectively, and *B* is the field strength.^[^ ^20^, ^42^ ^]^ In our measurements, the MR of NWs with *d* ≈ 233, 125, and 63 nm decreases unequivocally with the decrease in the core *d*. A log *MR* versus log *B* yields a straight line of slope 1.6 ± 0.03 for *d ≈* 233 nm and 1.4–1.3 for TaAs_2_ *d* ≈ 125 and 63 nm, respectively (inset, Figure 3b), whereas in a *d* ≈ 300 nm TaAs_2_ core, the slope was 1.8 ± 0.05 (inset, Figure 3a). Such a slope decrease suggests the weakening of semiclassical quadratic field dependence of MR in thinner NWs. Figure 3c summarizes the dependence of the maximal MR on TaAs_2_ *d* (red solid line is the linear fitting); the MR increases almost linearly with the increase in *d* and spanning from 47% (*d* ≈ 63 nm) to 2100% (*d* ≈ 420 nm). The decrease of the MR in thinner NWs can be explained by the correlation between the cyclotron orbit diameter with respect to the NW *d* in an external magnetic field. It has been shown in compensated semimetals, e.g., Bi and Sb NWs, that the MR exhibits a maximum at the field where the cyclotron radius becomes roughly equal to the NW's radius, and below this field, surface scattering dominates.^[^ ^46^, ^47^ ^]^ Therefore, thinner NWs would need a stronger field translated into a smaller cyclotron orbit to produce MR of the same magnitude. Notably, GMR observed in TaAs_2_ NWs of *d* in the range of 250–400 nm are among the highest in the reported trivial and topological semimetals/insulator NWs.^[^ ^25^, ^47^, ^48^, ^49^ ^]^ Note that the MR in TaAs_2_ NWs also shows a robust behavior against temperature, and decays very slowly up to 100 K (Figure 3d; and Figure S15a, Supporting Information), unlike in bulk phase_,_ where it drops orders of magnitude under the same conditions.^[^ ^22^ ^]^ A MR of 13%–15% was recorded in NWs of *d* ≈ 250–350 nm even at room temperature which are comparable or marginally superior to the values reported for bulk TaAs_2_^[^ ^21^ ^]^ (Figure 3d; and Figure S15a, Supporting Information).

The GMR in TaAs_2_ NWs can further be tuned by electrical gating, wherein the naturally grown SiO_2_‐shell works as an efficient intrinsic gate dielectric. Thus, the in situ grown shell around the TaAs_2_ core has a double benefit: it acts as a removable protective layer for electrical contact and a dielectric layer for gating. Gating experiments are presented in Figure S16 and the Supporting Information discussion.

The quantum oscillatory part of MR is characterized by subtracting the semiclassical background (Figure 3e; and Figure S15b, Supporting Information). Oscillatory features at 2 K are characteristic of the Shubnikov–de Hass (SdH) effect.^[^ ^22^ ^]^ The rapid periodic beating of the resistivity indicates Landau quantization of complex Fermi surfaces with different high‐mobility carriers. Unlike two major oscillation frequencies observed in bulk TaAs_2_,^[^ ^22^ ^]^ the FFT of oscillations in NWs manifest one major frequency peak at 53 T (Figure 3f). The corresponding Fermi surface area (*A*_F_) normal to the applied field was deduced employing the Onsager relation, *F* = (*Φ*/2*Π* ^2^)*A*_F_, wherein *F*, *Φ* = (*h*/2*e*), and *A*_F_ are the FFT peak position, flux quantum, and Fermi surface cross‐sectional area, respectively. Thus, the calculated *A*_F_ = 0.50 ×10^−2^ (Å^−2^) is in agreement with ARPES results on bulk TaAs_2_.^[^ ^24^ ^]^ Furthermore, the Lifshitz–Onsager quantization equation $A_{F}\frac{\hbar }{eB}=2\Pi \left(n+1/2+\beta +\delta \right)$ can be used to identify trivial (0) or nontrivial (*Π*) Berry phase, where *n* is Landau‐level index, 2*Πβ* is the Berry phase, and 2*Πδ* is a parameter linked to the Fermi surface curvature with its value *δ* = ±1/8 for a 3D Fermi surface (+1/8 for a minimal and −1/8 for a maximal cross‐section of the constant energy surface).^[^ ^3^, ^50^ ^]^ The inset in Figure 3f shows the Landau fan diagram, with integer (*n*) and half‐integer (*n* +1/2) indices representing Δ*ρ* maximum (peak) and minimum (valley), respectively. The linear dependence of Landau index *n* on 1/*B* gives *x*‐intercept = 1/2 + *δ* + *β* = 0.05 ± 0.01. Considering a minimal (*δ =* +1/8) and maximal (*δ =* −1/8) cross‐section yields *β* = −(0.57 ± 0.01) and −(0.325 ± 0.01), respectively, and thus a Berry phase (2*Πβ)* values of = −(*Π* ± 0.1) and −(0.65 *Π* ± 0.1), respectively. The occurrence of −(*Π* ± 0.1) Berry phase is a strong indication of the presence of Dirac/Weyl Fermions in a topologically nontrivial Fermi surface.^[^ ^3^, ^50^, ^51^ ^]^ The small variation in the obtained Berry phase for maximal cross‐section could be due to the strongly anisotropic Fermi surface^[^ ^51^ ^]^ of TaAs_2_.^[^ ^22^ ^]^

### 2.4 Anisotropic and Negative Magnetoresistance

**Figure 4:**
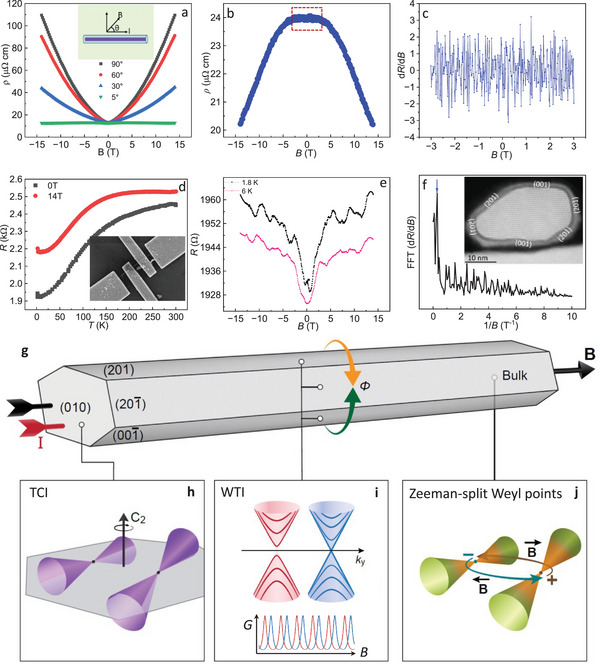
Anisotropic magnetoresistance, Aharanov–Bohm (AB) oscillations and intertwined topological states in TaAs_2_ NWs. a) Resistivity at 2 K as a function of magnetic field under different orientations of a NW (*d* ≈ 270 nm) with respect to the field direction; inset shows top‐view schematic presentation of NW‐axis rotation with respect to field. b) Negative MR in a NW (*d* ≈ 128 nm) under perfect longitudinal field configuration (*θ* ≈ 0°) at 2 K. c) MR oscillations/fluctuations for the same NW device in a field (dotted square in (b)) range (±3 T). d) Resistance as a function of temperature for the *d* ≈ 32 nm NW (inset: SEM image of device) in the absence and presence of 14 T filed at *θ* = 90°. e) Longitudinal MR and AB oscillations at 2 and 6 K in the same NW (*d* ≈ 32 nm) with a field aligned in the NW‐axis (*θ* = 0°). f) FFT spectrum of **e** obtained after nonoscillating MR background subtraction; inset shows TEM image of the same NW cross‐section with facets harboring weak topological insulator (WTI) states. g) Schematic representation of a TaAs_2_ NW with topologically nontrivial facets enabling quantum coherent surface transport in a longitudinal magnetic (**B**) and electric (**I**) field configuration (*θ* ≈0°). h) Topological‐crystalline‐insulator (TCI) state with a pair of C_2_‐symmetry protected overtitled type‐II Dirac cones on the (010) surface. Note that these surface Dirac cones can only be accessed at the two ends of NWs and thus cannot contribute to the measured magnetotransport reported here. i) A pair of type‐I Dirac cones (light red and blue) associated with the WTI state on the {001}, {201}, and {20$\bar{1}$} surfaces of the NW wall, quantized into discrete subbands (bright red and blue lines) by the quasi‐1D compact geometry of the NW; the inset shows a schematic representation of AB oscillation doublet peaks originating from the superposition of two phase‐shifted interference patterns arising from the two Dirac cones. j) Zeeman‐split pair of bulk Weyl cones with opposite chirality (“+” and “−”), with opposite directions of chiral charge pumping (green and brown arrows) controlled by the orientation of the electric and magnetic field, resulting in the observed longitudinal negative magnetoresistance (LNMR).

We next examined the magnetotransport in a TaAs_2_ NW (*d* ≈ 270 nm) under different angular orientations (*θ*) between the NW axis and field direction (inset, **Figure** **4**a). The MR shows a strongly anisotropic behavior, where the GMR decreases with decreasing the angle between the NW axis and field direction (Figure 4a; and Figure S17a, Supporting Information). A notable downward cusp of the resistivity near zero fields becomes shallow and broadens as the angle approaches *θ* ≈5°–6°, followed by a positive MR turning into a negative at higher fields (Figures S17b and S18a, Supporting Information). This angle dependence of the observed cusp (Figure S18b, Supporting Information) indicates a sign of 2D weak antilocalization (WAL), expected from the topological surface states.^[^ ^52^ ^]^ In a relatively thinner NW (*d* ≈ 128 nm) under a perfectly longitudinal field configuration (*θ* = 0°), an unambiguous longitudinal negative magnetoresistance (LNMR) was observed in the entire measured field range (Figure 4b). The field dependence of LNMR fits equation *ρ = ρ*_0 –_ 0.020(*B*)^2^ wherein *ρ, ρ*_0_, and *B* represent field‐dependent resistivity, zero‐field resistivity, and field strength respectively (Figure S19, Supporting Information). LNMR of NWs aligns with a similar effect reported in bulk TaAs_2_.^[^ ^23^ ^]^ The LNMR in TaAs_2_ and other TX_2_ analogs potentially originate from the chiral charge pumping (Weyl fermions transport) between the Weyl nodes of opposite chirality induced by Zeeman splitting of bulk bands (Figure 4j).^[^ ^7^, ^43^, ^53^ ^]^ The presence of topologically nontrivial Fermi surface with *Π* Berry phase (indicative of Dirac/Weyl fermions) in TaAs_2_ NW is in support of this argument.^[^ ^54^ ^]^

### 2.5 Magnetoresistance Oscillations/Fluctuations in a Longitudinal Field

To probe the coherent electron transport linked to surface Dirac fermions,^[^ ^26^ ^]^ oscillations were measured in the same NW (*d* ≈ 128 nm). Multiple peaks with a period close to 0.1 T were recorded (Figure 4c); however, the FFT spectrum (Figure S20, Supporting Information) obtained by subtracting the LNMR background (d*R*/d*B*) did not show a clear periodicity. This can be a sign of universal conductance fluctuations, which occur due to the bulk semimetallic states coexisting with the surface states,^[^ ^24^ ^]^ resulting in a lack of a perfect electron coherent path at the measured temperature.

To further enhance the contribution of the surface and decrease that of bulk electronic states, a device was fabricated on a much thinner NW (*d* ≈ 32 nm), with metallic conductivity and no apparent electrical transitions even in a transversal magnetic field of 14 T (Figure 4d), indicating a situation of dominant surface transport due to much smaller NW diameter with respect to cyclotron orbit.^[^ ^46^ ^]^ The absence of an LNMR further confirmed that transport here is dominated by surface states.^[^ ^46^, ^47^ ^]^ Note that this thinner TaAs_2_ NW not only shows sign‐reversal of MR but also manifests much stronger longitudinal MR oscillations, indicating a significant expression of TSS transport (Figure 4e). The period of oscillation (Δ*B*) remains roughly constant in the entire field range with the oscillation amplitude decaying at higher temperature. The FFT d*R*/d*B* gives a major oscillation peak at ≈0.25 T^−1^, associated with the Δ*B* ≈4.1 T (Figure 4f) with no apparent second harmonic signal. The AB oscillation period that one would expect for a TI NW of this diameter should be Δ*B* = *Φ*_0_/*A*, where *Φ*_0_ = *h*/*e* is the flux‐quantum, *h* is Plank's constant, *e* is elementary charge, and *A* is the NW cross‐section area.^[^ ^26^ ^]^ Considering that the NW cross‐section measured by TEM is *A* = 0.5 × 10^−15^ m^2^ (Figure 4f, inset), the oscillation period thus expected would be Δ*B* = 8.3 T. This value is about 2 times larger than the experimental value Δ*B* ≈4.1 T, corresponding to a Δ*B* period of *h*/2*e* (divided by *A*) rather than *h*/*e*.

The doubling of the oscillation frequency can a priori be attributed to two effects: i) Altshuler–Aronov–Spivak (AAS) oscillations, known to occur in diffusive metallic cylinders and have a period of *h*/2*e*,^[^ ^26^ ^]^ and ii) a double AB oscillation pattern stemming from the fact that our wire is a WTI^[^ ^6^, ^7^, ^8^ ^]^ and not a strong TI, and hence it has two surface Dirac cones instead of one, each contributing its own AB oscillation pattern. Although ASS oscillations cannot be ruled out at this stage, we can expect them to be a less likely mechanism in our wire, for several reasons. First, the measured conductance is 5.1 × 10^−4^ S (Figure 4e; and Figure S22, Supporting Information), which is ≈13 times higher than the conductance quantum *G*_0_ = *e* ^2^/*h*. Such large conductance, especially for a thin NW whose magnetoresistance indicates that transport is dominated by surface states, is indicative of ballistic/quasi ballistic rather than diffusive transport. The fact that NWs reported here shows a higher conductivity than bulk TaAs_2_ (Figure S11, Supporting Information) also supports coherent surface transport. The reported mean free path for TaAs_2_ is 400 µm,^[^ ^23^ ^]^ whereas the channel length of the device where oscillations were recorded is 570 nm, further making the case of ballistic or quasiballistic transport rather than a diffusive transport. Second, the TaAs_2_ NWs studied in this work possess a highly crystalline chemically protected surface with no traces of disorder (Figure 4f, inset, and structural characterization), and are p‐doped (Figure S16 and the Supporting Information discussion), resulting in conductance much higher than *e* ^2^/*h* and a maximum of magnetoconductance at zero flux (Figure S22, Supporting Information), compatible with AB oscillation.^[^ ^55^ ^]^ Third, AAS oscillations are expected to become more pronounced in thicker NWs.^[^ ^56^ ^]^ However, a thicker 128 nm diameter NW device (Figure 4c; and Figure S20, Supporting Information) did not show any oscillations with a well‐defined period. Fourth, detecting a signal contributed solely by AAS oscillations is rare in the local configuration of a conventional four‐probe as ours (Figure 4d, inset), and is usually observed only in nonlocal configurations.^[^ ^57^ ^]^

A double AB oscillation pattern (ii), on the other hand, would be consistent with the two Dirac cones of a WTI surface instead of the single Dirac cone of a TI, as schematically represented in Figure 4i. WTIs are predicted to feature a pair of type‐I Dirac cones on the surfaces relevant to our NW configuration,^[^ ^6^, ^8^ ^]^ namely parallel pairs of {201}, {001}, {20$\bar{1}$} facets (Figure 4f, inset and Figure 4g,i). The two Dirac cones of the WTI reside on different points of the surface Brillouin zone and can thus generate two AB oscillation patterns with the same period, but a phase shift between them (see Figure 4i; and Figure S21 and the Supporting Information text for a theoretical model of this effect).^[^ ^58^ ^]^ Since each AB oscillation pattern has a period *h*/*e*, a superposition of the two AB oscillation patterns can give the appearance of a *h*/2*e* period, as measured in our device (Figure 4e), instead of *h*/*e*. This doubling should be most pronounced when the two oscillations are shifted by half of their period. According to our model, the shift and the period scale differently with the cross‐section area. Therefore, the AB doubling will be either most pronounced or absent in NWs having specific cross‐section areas *A*. A NW having a random cross‐section area will most likely show a certain extent of doubling (as we observed in Figure 4e), even if not maximal (see the Supporting Information for a more detailed explanation).

In quantitative terms, a conductance of ≈13*G*_0_ suggests that 13–14 surface sub‐bands reside below the chemical potential. Considering that the sub‐band *k*‐spacing (δ*k*) is the inverse of the radius, namely δ*k =* (π/*A*)^1/2^, and the calculated Fermi velocity (*v*_F_) is 3 × 10^5^ ms^−1^,^[^ ^24^ ^]^ the energy gaps (δ*E* = *ℏv*_f_δ*k*) between the sub‐bands quantized by the quasi‐1D geometry are 16 meV. If the sub‐bands contributing to this conduction stem from one or two Dirac cones, as expected from a TI or a WTI, respectively, this would be consistent with a Fermi energy difference from the Dirac cones of 200 or 100 meV, respectively. The latter value is very close to that reported for this energy difference in TaAs_2_, namely 110 meV.^[^ ^24^ ^]^ This quantitative consistency further supports ballistic conduction by a WTI surface. Systematic corroboration of this effect in NWs of TaAs_2_ and other TX_2_ materials with well‐defined topologically nontrivial facets will pave the way to understanding and utilizing these topologically protected electrons in quantum nanotechnology.

## 3 Conclusions

We synthesized NWs of a topological semimetal (TSM) TaAs_2_ with intertwined topological states harboring rich electronic band topologies. These NWs possess a unique core–shell structure, with an amorphous SiO_2_ shell encapsulating the highly crystalline TaAs_2_ core, protecting it from oxidation or any other damage, giving remarkable ambient stability, and can be locally etched conveniently and selectively prior to electrode deposition, while leaving the rest of the NW encapsulated. The in situ grown shell not only protects the rich topological surface but also works as an all‐around gate dielectric for tuning the Fermi energy. These high‐quality NWs exhibit conductivity up to 15 times superior to the bulk TaAs_2_ with an impressively high ampacity of 3–4 mA under ambient conditions desirable for thermoelectrics and circuit interconnects. Introducing an external magnetic field perpendicular to the nanowire axis <010> gives rise to an metal‐to‐insulator (MI) transition near room temperature, which is four times higher than the reported transition temperature in bulk phase TaAs_2_. Further, at *T* ≤ *T*_i,_ the insulating state is replaced by a metallic surface conduction mode, resulting in a nontrivial insulator‐to‐metal (IM) transition associated with topological surface states (TSS). The TaAs_2_ NWs maintain TSS transport features at remarkably high temperatures, which is technologically important for spintronic devices and qubits. Furthermore, a GMR of the order of 10^3^ increasing linearly with the NW core diameter has been achieved. Such a nonsaturating GMR in NWs could have applications in magnetic switches, memory devices, and sensors. The SdH oscillations and the Landau fan diagram demonstrate the topological Fermi surface hosting Dirac fermions. The LNMR in the TaAs_2_ NW supports the origin of a chiral anomaly due to Zeeman field‐induced Weyl points. The observation of a double AB oscillation pattern, potentially due to the occurrence of a pair of Dirac cones associated to the WTI state, opens the path to further exploration of a coherent WTI surface. We show that TaAs_2_ NW with a thin protective shell of SiO_2_ reported in this work serves as an excellent system to observe rich transport phenomena originating from intertwined electronic band topologies, namely a pair of type‐I surface Dirac cones linked to WTI state, type‐II Dirac cones due to TCI state, and Weyl points. Figure 4g–j summarizes the intertwined topological phases hosted by the different facets of NW and their bulk, which manifest themselves in the presented results. The incorporation of superconductivity coexisting with rich TSS in TaAs_2_ NWs can potentially provide a platform to generate Majorana fermions for topological quantum computers.^[^ ^59^ ^]^ The protective SiO_2_ around the TaAs_2_ NWs makes them chemically robust and compatible with CMOS technology. Furthermore, the synthesis demonstrated here could be extended to obtain surface‐clean, naturally encapsulated single‐crystalline NWs of other dipnictides, like TaP_2_, NbP_2_, TaSb_2_, and NbSb_2_, VP_2_, VAs_2_, VSb_2_ thus enabling the exploration of rich topological phases, exotic physical phenomena, and their potential applications in quantum nanotechnology as spintronics, magnetoelectric sensors, and quantum computing.

## 4 Experimental Section

### Synthesis of Nanowires

TaAs_2_ NWs were synthesized inside a vacuum‐sealed quartz ampule. The ampule was designed to have spatial isolation of 21–22 cm between the precursor activation and nanowire growth zone. In the precursor activation zone, a molar ratio of TaCl_5_ and excess arsenic (As) (TaCl_5_‐to‐As ratio ranging from 1:1 to 1:4) powders were loaded in separate quartz cups. In the growth zone, 7 × 7 mm silicon (Si with native oxide) and different crystallographic cut (R, M, C, and AM (annealed M‐plane)) α‐Al_2_O_3_ substrates were placed horizontally (Figure 1a). Considering the air and moisture sensitivity, precursors were handled and loaded in the quartz ampule inside a nitrogen glovebox and transferred to a vacuum sealing system under protection. This sealed ampule was placed horizontally in a two‐zone tube furnace wherein the precursor zone was maintained at 600 °C, and the growth zone was maintained at 1020 °C for 20 min, followed by the immediate quenching of reaction to room temperature. As a result of the synthesis, the growth substrates were coated with a high yield of NWs along with some NBs. High‐magnification SEM and TEM imaging showed that NWs and NBs are uniformly encapsulated in a thin amorphous shell of SiO_2_, producing a unique core–shell structure. Control experiments confirmed that the SiO_2_ shell on TaAs_2_ NW grows from a chemical vapor reaction of Si substrate (with native oxide layer) with liberated Cl_2_ vapor (Please refer to the Supporting Information for more details on the synthesis and plausible growth mechanism). NW of other TX_2_ analogs with all‐around thin‐dielectric SiO_2_ shells were also obtained employing similar synthetic conditions, confirming the generality and reproducibility of this unique synthetic methodology. In synthesis attempts wherein the Si substrate with native oxide layer was replaced by Si/SiO_2_ (with 300 nm thermal oxide layer), very low yields were observed of NWs.

### Characterization

The morphology of nanowires/nanoribbons was examined in a scanning electron microscope (Sigma 500 SEM, Zeiss). The core–shell structure of NWs/NRs was examined in a high‐magnification SEM (Sigma 500 Zeiss) at an accelerating voltage of 10 KV and further confirmed using TEM (Talos F200X G2 TEM). Thin electron‐transparent cross‐sections of nanowire/nanoribbon were obtained by focused‐ion‐beam (FBI, FEI Helios 600, Dual‐beam microscope). Crystal structure, atomic resolution imaging, EDS mapping, and interface imaging were done using a high‐resolution transmission microscope (HR‐TEM, Themis‐Z). Raman spectrum of the samples was recorded using a micro‐Raman spectrometer (Horiba LabRAM HR evolution). A 532 nm green laser was aligned and focused on a single NW/NR specimen under an optical resolution of X150.

### Device Fabrication and Transport Measurements

Four‐terminal devices were fabricated from NWs on α‐Al_2_O_3_ or Si/SiO_2_ (300 nm thermal oxide layer) substrates. In the case of Si/SiO_2_ substrate, a 20 nm of HfO_2_ layer was deposited using atomic layer deposition (ALD). NWs were transferred on the substrate and spin‐coated with a positive tone photoresist (S1813) or electron beam resist (PMMA‐A5). This was followed by a laser‐assisted or electron‐beam lithography, to open four‐hole windows (size ≈1–5 µm) on the TaAs_2_‐SiO_2_ core–shell NW. The SiO_2_‐shell was locally etched through these windows using a buffered oxide etchant (BOE 6:1; 6:1 volume ratio of 40% NH_4_F in H_2_O to 49% HF in H_2_O) for 30–60 s to expose the TaAs_2_‐core and immediately an electron beam evaporation of Cr/Au (10/250 nm or 10/150 nm) was done on the locally etched area of NW to make the ohmic contacts with TaAs_2_ core, while maintaining the SiO_2_‐shell on the rest part of the NW (Figure S7, Supporting Information). Use of ALD‐HfO_2_ capping layer on Si/SiO_2_ protects the substrate against BOE etching. The magnetotransport measurements were performed in a Quantum Design 14 T PPMS Dynacool system, using the electrical transport and the resistivity modes. These options generate either a DC or an oscillating low‐frequency current and measure the voltage across the device using a four‐probe wiring scheme. Currents of up to 1 mA were used, with frequencies around 0.5 Hz in the electrical transport option. The angle‐dependent measurements were done using Dynacool's horizontal rotator option. Back and top‐gate voltage was applied using a Keithley 2450 source meter.

## Conflict of Interest

The authors declare no conflict of interest

## Supporting information



Supporting Information

## Data Availability

The data that support the findings of this study are available from the corresponding author upon reasonable request.
