# Supplementary material for: Intertwined Topological Phases in TaAs2 Nanowires with Giant Magnetoresistance and Quantum Coherent Surface Transport
Source: Adv Mater. 2025 Mar 27;37(28):2418279. doi: 10.1002/adma.202418279 (PMC12271997; doi:10.1002/adma.202418279)
Supplement: Supplementary file 1 — Supporting Information [file ADMA-37-2418279-s001.pdf]

ADVANCED MATERIALS

Supporting Information

for *Adv. Mater.*, DOI 10.1002/adma.202418279

Intertwined Topological Phases in $TaAs_2$ Nanowires with Giant Magnetoresistance and Quantum Coherent Surface Transport

Anand Roy, Anna Eyal, Roni Majlin Skiff, Barun Barick, Samuel D. Escribano, Olga Brontvein, Katya Rechav, Ora Bitton, Roni Ilan and Ernesto Joselevich**

Supporting Information for

Intertwined Topological Phases in $TaAs_2$ Nanowires with Giant Magnetoresistance and Quantum Coherent Surface Transport

Anand Roy[a*], Anna Eyal[b], Roni Majlin Skiff,[c] Barun Barick[d], Samuel Díaz Escribano[d], Olga Brontvein[e], Katya Rechav[e], Ora Bitton[e], Roni Ilan[c] and Ernesto Joselevich[a*]

[a]Department of Molecular Chemistry and Materials Science, Weizmann institute of science, Rehovot 7610001, Israel; [b]Physics department, Technion, Haifa, 32000, Israel; [c]Raymond and Beverly Sackler school of Physics and Astronomy, Tel Aviv 69978, Israel [d]Department of Condensed Matter Physics, Weizmann Institute of Science, Rehovot 76100, Israel. [e]Chemical Research Support, Weizmann Institute of Science, Rehovot 76100, Israel. e-mail: ernesto.joselevich@weizmann.ac.il, anand-kumar.roy@weizmann.ac.il.

Growth conditions for core-shell $TaAs_2$-SiO_2 NWs and possible mechanism

Several synthetic strategies and many attempts were carried out to achieve the growth conditions to reproducibly obtain the very long (at the best-optimized synthesis condition, NWs have a length from a few tens to a few hundreds of microns) highly crystalline, core-shell NWs reported in the manuscript. The successful syntheses of core-shell $TaAs_2$-SiO_2 NWs were achieved inside a vacuum-sealed quartz ampule as shown in **Figure 1a**, under a large window of growth temperature ranging from 915 to 1035 °C, and under $TaCl_5$-to-As molar ratio ranging from 1:1 to 1:4.

A careful analysis of different synthetic strategies and attempts can provide understanding of plausible growth mechanism. For instance, replacing $TaCl_5$ with $TaBr_5$ under similar growth conditions also yields NWs, but the aspect ratio of the synthesized core-shell nanowires and the yield are a far-cry from the nanowires reported in the main text. Fine tuning of the synthesis and its optimization, e.g., by varying the amount of $TaBr_5$, growth time, and temperature, could in the future lead to high quality and high yield of the core-shell NWs as reported in the manuscript using $TaCl_5$.

The small amounts of $TaCl_5$ (2-4 mg) used in the role of a transport agent in the presence of Ta and As powders in the precursor zone, do not yield any NW. This experiment suggests it is essential to have a Ta precursor with low evaporation temperature like $TaCl_5$ to facilitate the chemical vapor formation.

We have also conducted synthesis using Ta and As powders or TaAs powder in the presence of different concentrations of iodine (I_2) as a transport agent under similar synthetic conditions (in the presence of Si/Au and α-Al_2O_3 substrates in the growth zone), but NWs as reported in the manuscript were not observed, further indicating the need of tantalum halide precursors.

Furthermore, surface-assisted CVD (SACVD) using a Ta foil in the growth zone, which is known to produce Ta chalcogenides NWs,[1] was employed under two different synthetic conditions. In the first approach (a), synthetic conditions were similar to those reported in the manuscript but Si substrates in the growth zone were replaced by Ta foil of the same size. In the second approach (b), $TaCl_5$ in the precursor zone was replaced by Ta powder. Both synthetic approaches yielded no NWs. These experiments suggest the need for both $TaCl_5$ (in the precursor zone) and Si (in the growth zone) to produce NWs.

From these series of experiments we can conclude that a bare presence of Ta and As precursors in reported synthetic conditions does not produce $TaAs_2$ NWs or in fact any NWs. It is crucial to have $TaCl_5$/$TaBr_5$ and As (anywhere from 1:1 to 1:4 mmol ratio) in the precursor zone and Si or Si/Au (Au ~ 1 to 5 nm) substrates in the growth zone to achieve the growth of core-shell $TaAs_2$-SiO_2 NWs on different cut α-Al_2O_3 substrates placed a few mm to cm from the Si substrates. The fact that the $TaAs_2$-SiO_2 core-shell NWs do not form on the Si substrate but on the spatially separated α-Al_2O_3 substrates as well as on the inner wall of the ampule in the growth zone suggests that the Si plays a critical role in generating the volatile precursor for the formation of the SiO_2 shell. We suggest that Cl_2 vapor arising from partial decomposition of $TaCl_5$ could react with the Si (or its native oxide) to form $SiCl_4$ vapor, which can then react with oxygen traces (perhaps released from the native oxide layer of Si substrates) to form the amorphous SiO_2 shell around the $TaAs_2$ cores that grow on the α-Al_2O_3 substrates and ampule inner wall. This reaction mechanism hereby suggested could be represented by the following processes:

$2\ TaCl_5(s) \rightarrow 2\ Ta(s) + 5\ Cl_2(g)$ (in the $TaCl_5$ powder boat)

$SiO_2(s) + 2\ Cl_2(g) \rightarrow SiCl_4(g) + O_2(g)$ (on the Si substrate with native oxide)

$SiCl_4(g) + O_2(g) \rightarrow SiO_2(s) + 2\ Cl_2(g)$ (on the surface of the $TaAs_2$ NWs grown on the α-Al_2O_3 substrates and ampule inner wall in the growth zone)

Note that, decreasing or increasing the growth time from 20 minutes only affects the $TaAs_2$ core diameter but has no effect on the thickness of the SiO_2 shell (shell always has a thickness of 60 to 80 nm) whatsoever. This suggests that the SiO_2 shell does not grow simultaneously with the $TaAs_2$ core. In fact, we surmise that the amorphous SiO_2 shell grows while the sample is cooling down from the growth temperature to room temperature, and hence, the shell thickness does not change with the $TaAs_2$ core growth time.

Control experiments wherein the sample was not exposed to the ambient atmosphere before SEM imaging and was exposed to the ambient atmosphere for the entire duration of opening of the sealed ampoule to the SEM sample preparation were undertaken. These experiments did not show any noticeable difference in the SiO_2-shell thickness, confirming that the SiO_2 shell forms *in situ* and is not affected by the interaction with ambient oxygen.

To understand why bare $TaAs_2$ NWs without a SiO_2 shell do not form in the absence of a Si substrate, we did a control experiment under similar synthetic conditions but in the absence of As powder and probe if $TaSi_2$ NWs act as a growth precursor for the $TaAs_2$ NW. However, we did not notice any NW growth, confirming that $TaSi_2$ is not the precursor for the $TaAs_2$ NW growth.

The fact that we did not observe growth of $TaAs_2$ NWs in the absence of Si substrates indicates the possibility of silicon-tantalum-arsenic containing species acting as an intermediate for the growth of $TaAs_2$ NWs, although no evidence of such intermediate could be found so far. Additionally, the fact that encapsulation of $TaAs_2$ NW core in a SiO_2 shell is highly selective (all the NWs are uniformly encapsulated in nearly the same thickness of SiO_2 shell) indicates that the core-shell growth mechanism is locally controlled, resembling the one reported in the reference.[2] Probing the exact mechanism by *in situ* growth and characterization inside an SEM or TEM, as we have performed for other materials[3] call for an interesting dedicated investigation in the future.

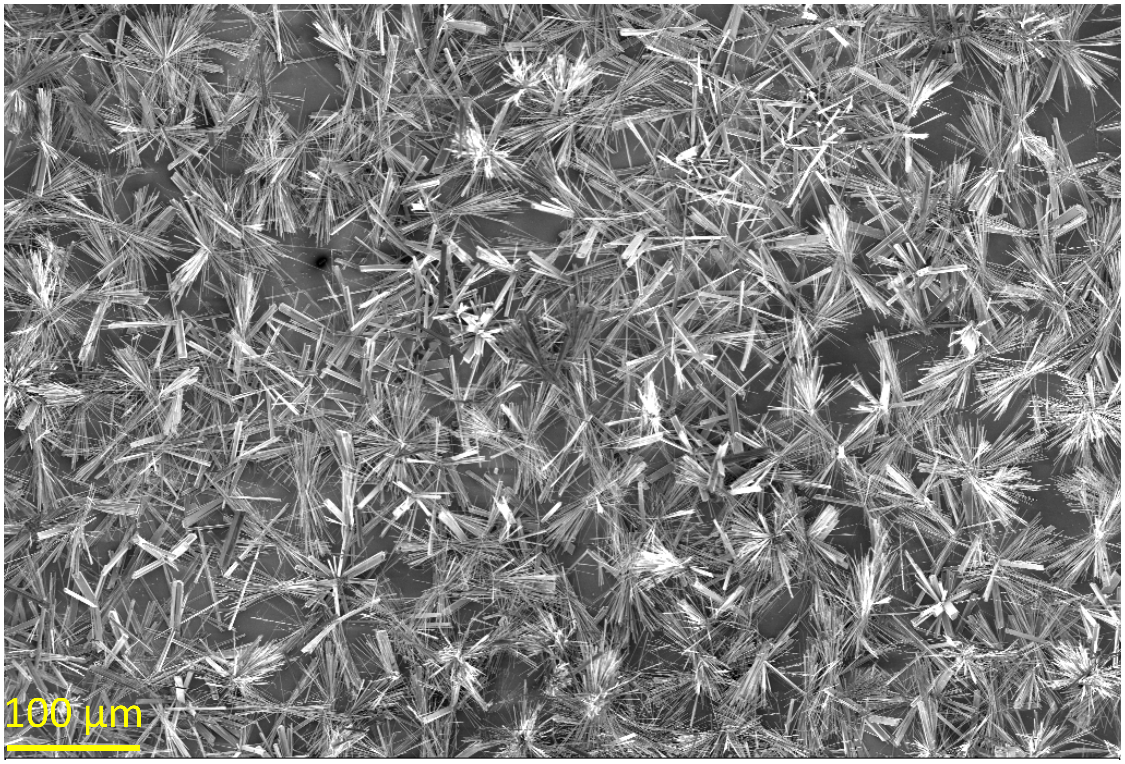

Figure S1. Large-area SEM image showing high yield of $TaAs_2$ nanowires (NWs) with some nanobelts (NBs) on a α-Al_2O_3 (sapphire) substrate.

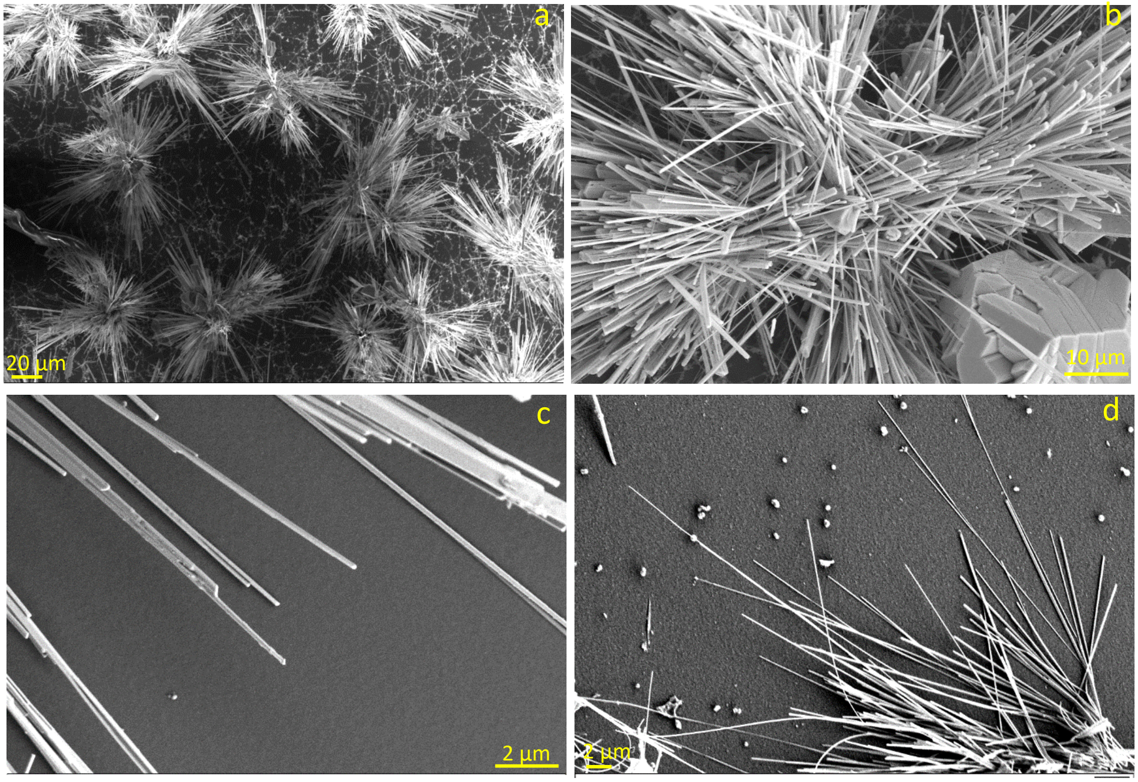

Figure S2. SEM images of $TaAs_2$ NWs grown of different cut (R, C, and annealed-M) α-Al_2O_3 substrates.

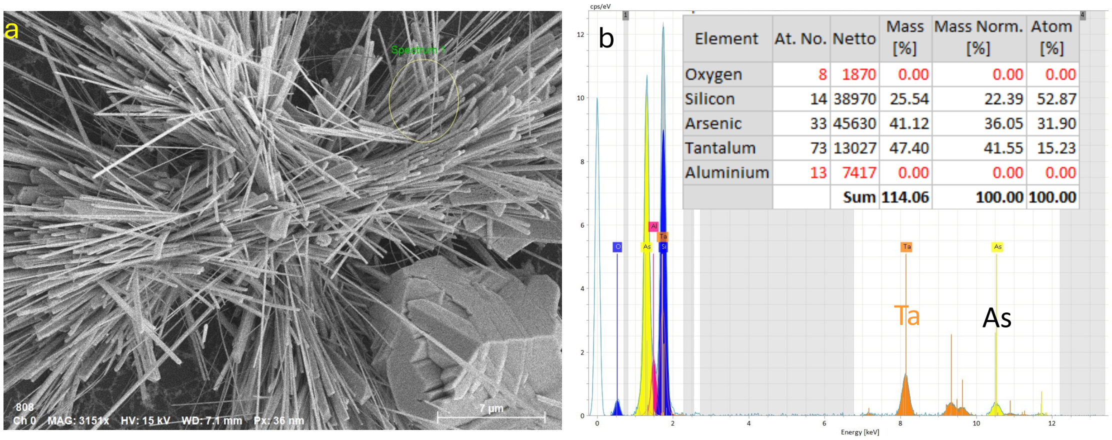

Figure S3. (a) SEM image showing an ensemble of NWs from which the EDS spectrum was measured. (b) EDS spectrum obtained from (a) showing signals of Ta, As, Si, and O atoms. The inset in (b) presents the atomic ratio of elements calculated from the obtained spectrum. The silicon signal originates from the SiO_2 shell.

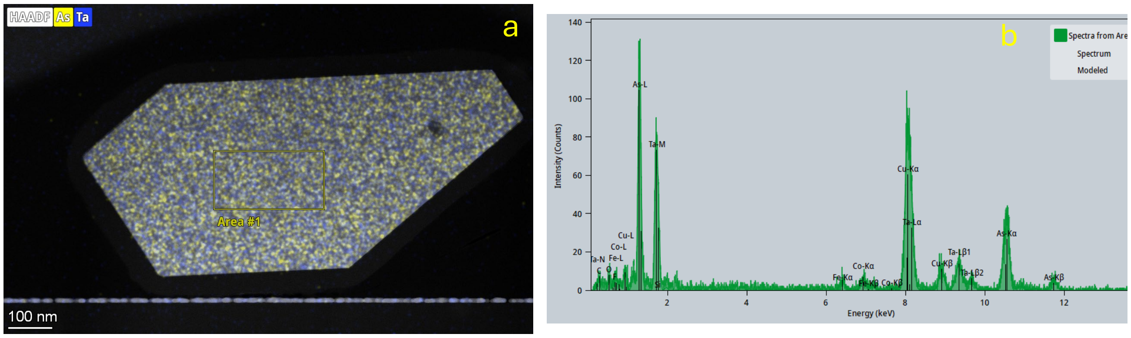

c

z	Element	Family	Atomic fraction (%)	Atomic error (%)	Fit Error (%)
33	As	K	66.08	2.78	0.76
73	Ta	L	33.92	2.78	0.60

Figure S4. (a) STEM image showing the cross-section of $TaAs_2$ core produced by focused ion beam (FIB), (b) Corresponding EDS spectrum obtained from the $TaAs_2$ core shows Ta and As signals, (c) Calculated atomic percentages of Ta and As from the core shows nearly 2:1 As : Ta ratio.

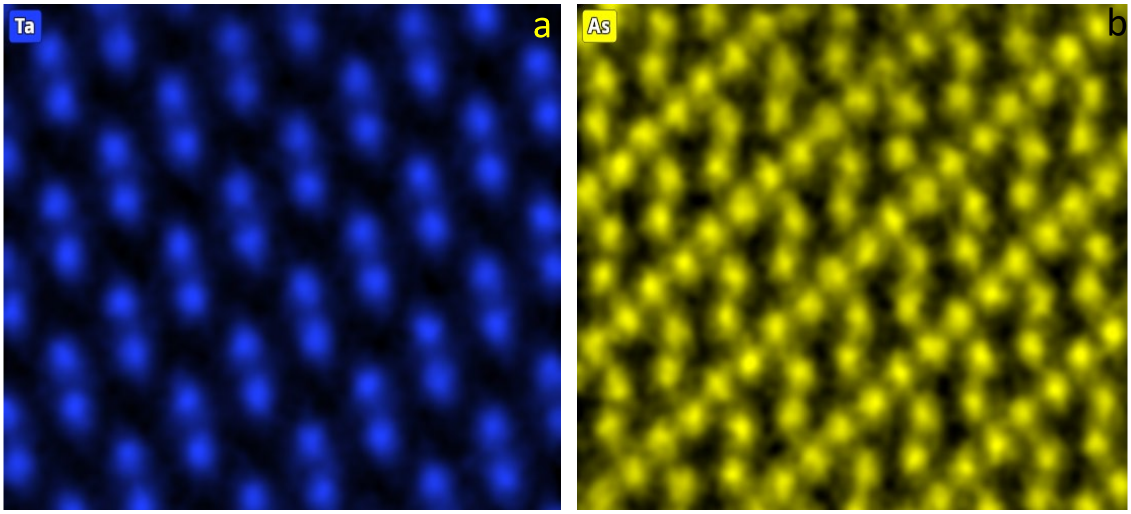

Figure S5. Atomic resolution EDS mapping of $TaAs_2$ NW cross-section showing lattice arrangement of (a) Tantalum (Ta), and (b) Arsenic (As) atoms.

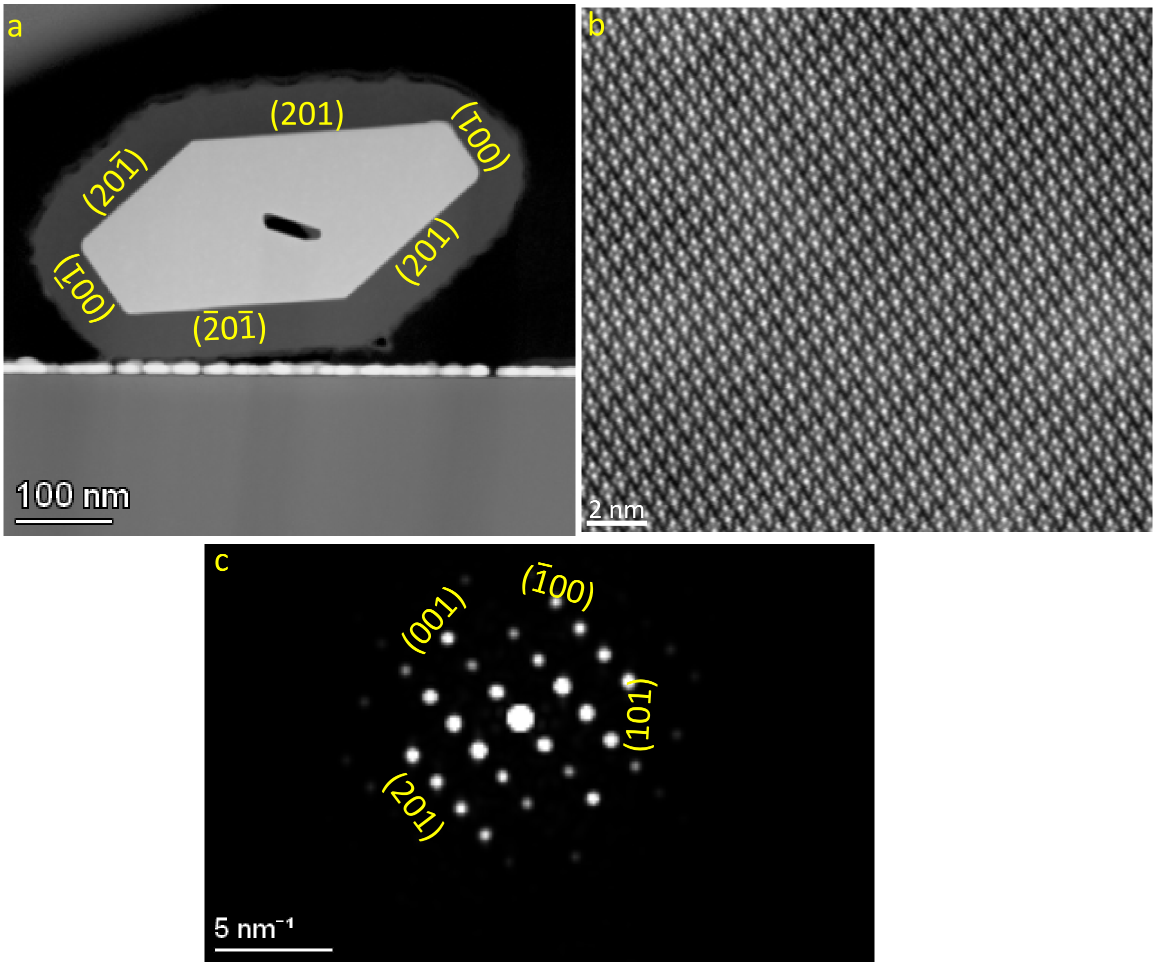

Figure S6. (a) HAADF-STEM image of a NW cross-section showing $TaAs_2$-SiO_2 core-shell structure. (b) HAADF-STEM atomic resolution image from the NW core showing highly ordered $TaAs_2$ crystal. (c) FFT pattern recorded from the central area in (b) showing single-crystalline structure of NW core with lattice planes of $TaAs_2$.

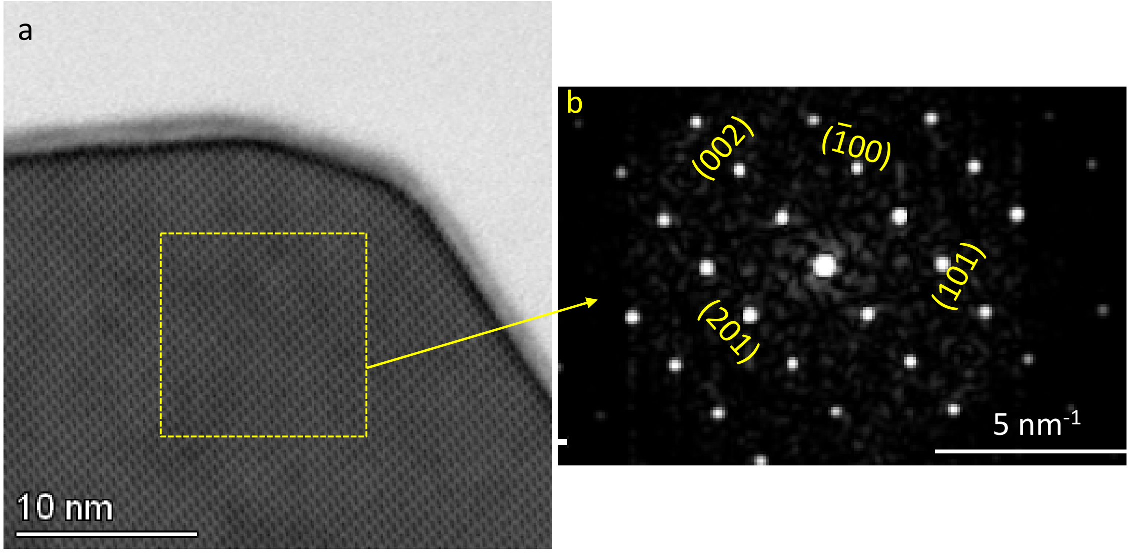

Figure S7. (a) High-resolution BF-STEM image from the interface region presenting highly ordered $TaAs_2$ core encapsulated with an amorphous SiO_2 shell. **b** The FFT pattern recorded from the square-box area manifesting single-crystalline structure of the $TaAs_2$ surface.

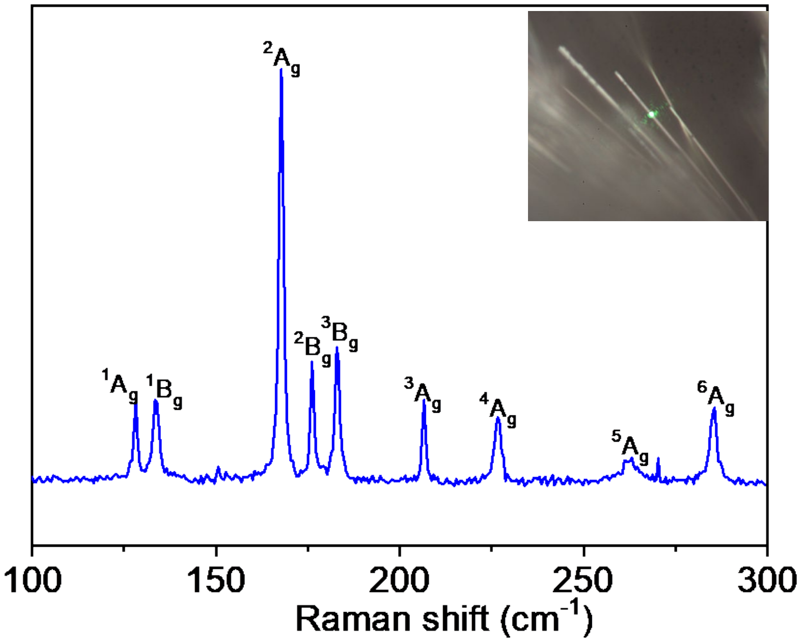

Figure S8. Room temperature Raman spectrum of $TaAs_2$ NW under a 532 nm (green) laser excitation. The peaks have been assigned based on a reported spectrum from bulk $TaAs_2$. Inset shows an optical image of a NW under laser excitation.

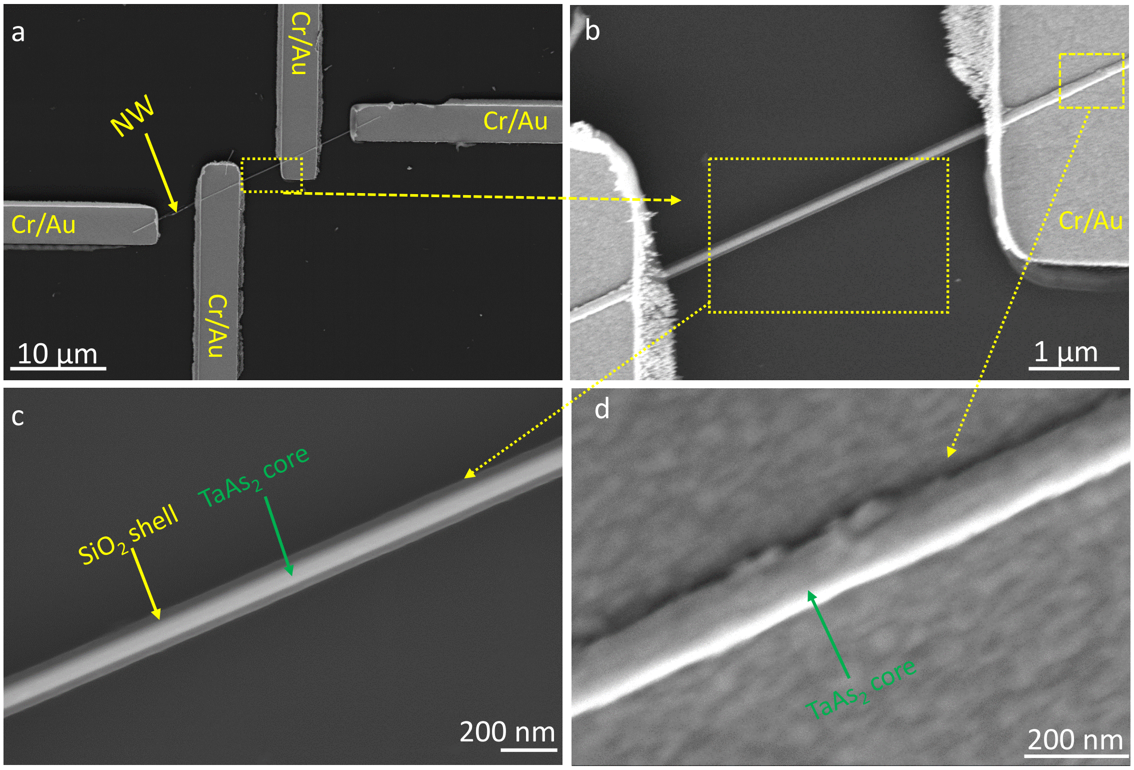

Figure S9. (a) SEM Image showing a typical four-terminal NW device. (b) low, and (c) high-magnification images showing in between the electrodes (Cr/Au) non etched part of NW, wherein $TaAs_2$ core encapsulated in a SiO_2 shell can be seen, (b) low and (d) high magnification images showing part of NW wherein SiO_2 shell was selectively etched after opening the lithography window to make an Ohmic (electrical) contact between $TaAs_2$-core and electrodes (Cr/Au:10/250 or 10/150 nm).

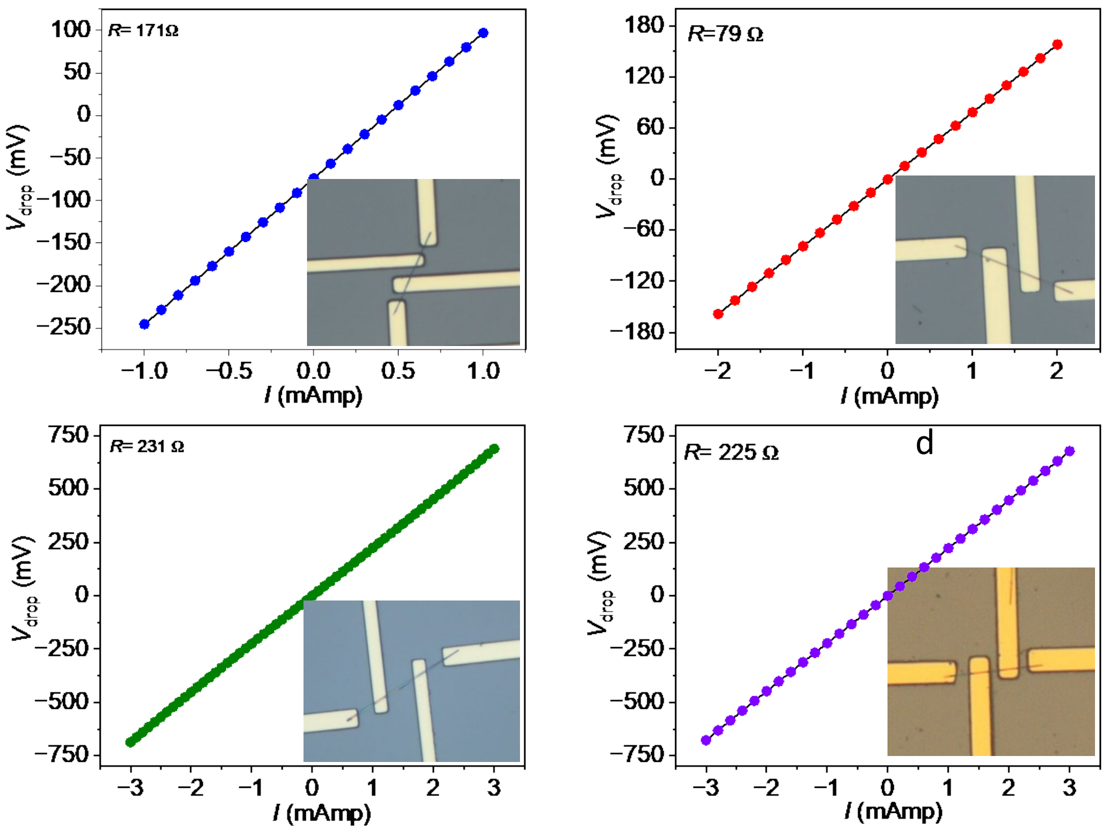

Figure S10. Ambient condition four-probe current-voltage (I-V) curves of different core diameter $TaAs_2$ NW devices under applied dc-currents (1 to 3 mA). Insets show optical images of NW-devices.

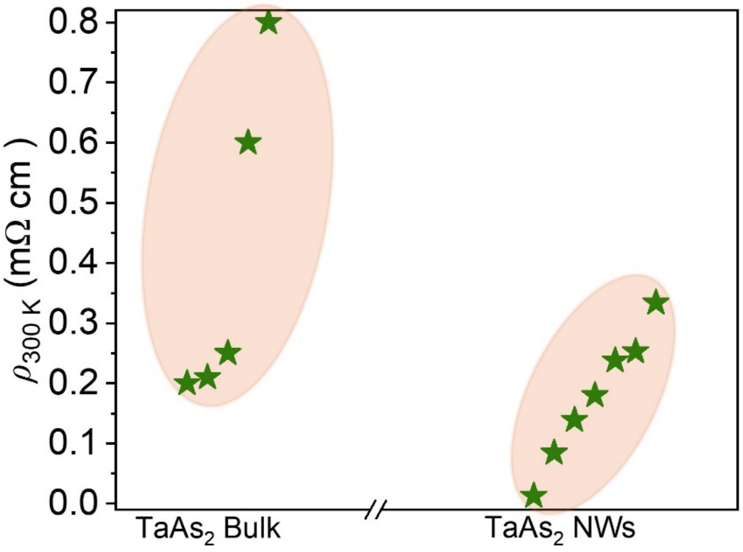

Figure S11. Room temperature resistivity values of $TaAs_2$ NWs with respect to the values for $TaAs_2$ bulk-single crystals. The resistivity values of bulk $TaAs_2$ have been taken from reported articles.[4-7]

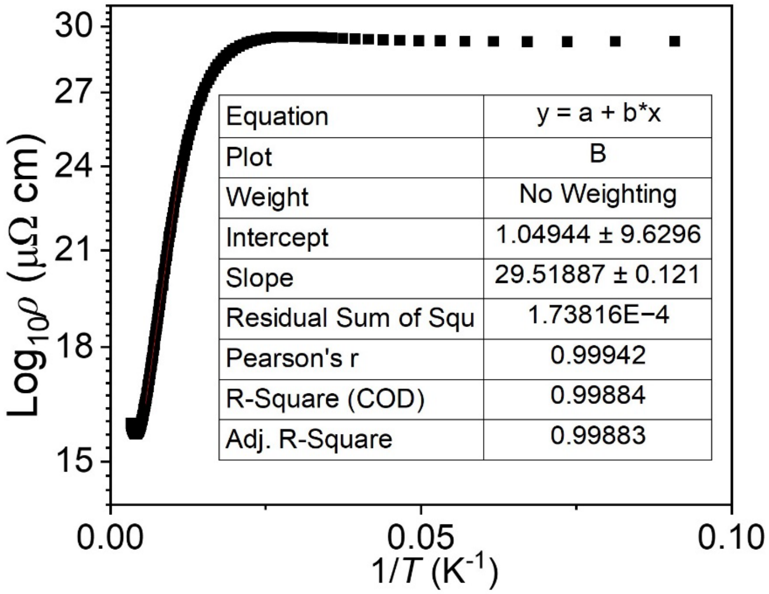

Figure S12. A linear fit of insulating state resistivity $\log_{10}\rho$ as a function of T^{-1} employing Arrhenius equation for a 300 nm core diameter $TaAs_2$ NW.

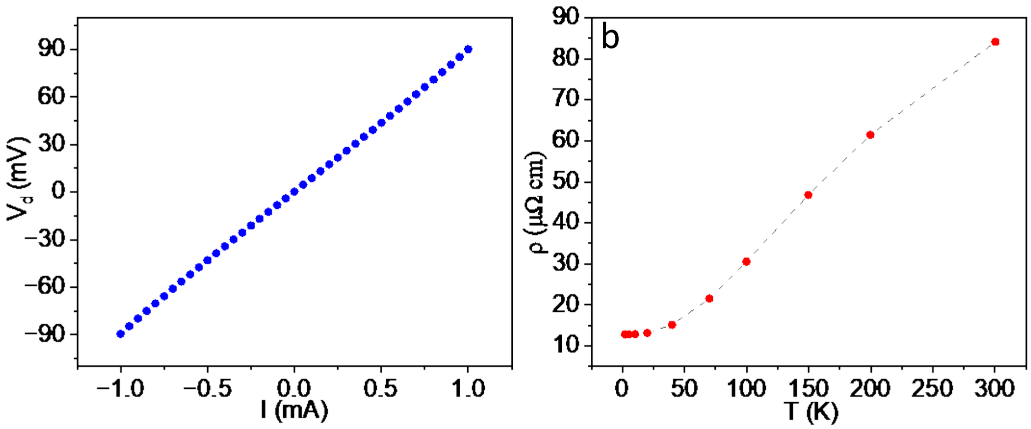

Figure S13. (a) Room-temperature I-V curve, (b) resistivity as function of temperature in a $TaAs_2$ NW with core diameter ~270 nm.

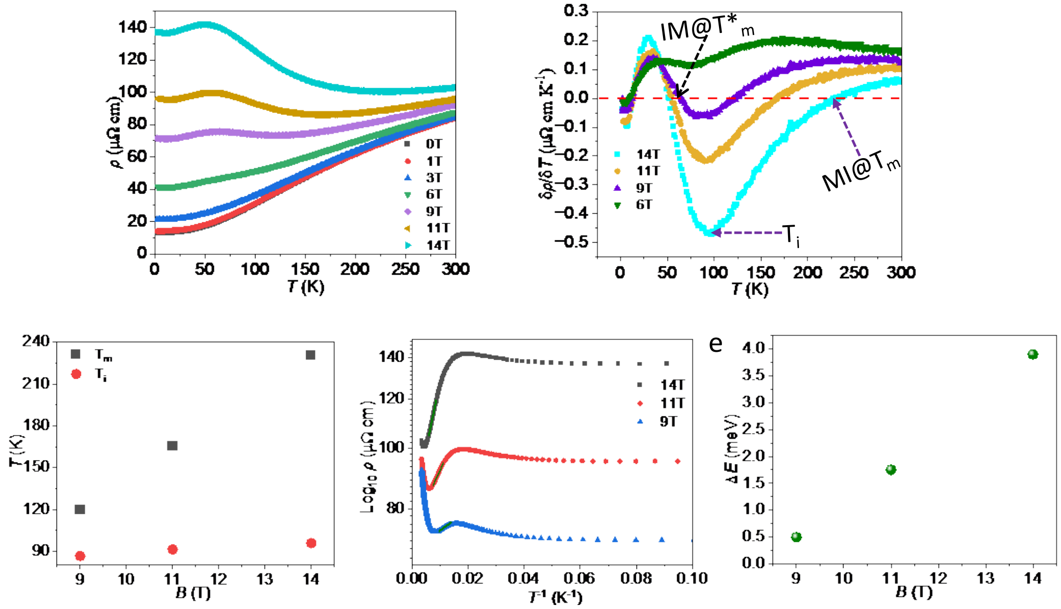

Figure S14. Magnetotransport properties in a $TaAs_2$ NW device with ~ 270 nm core diameter. (a) Resistivity as a function of temperature (RT) under increasing filed of 0 to 14 T. (b) The first derivative of resistivity $\delta\rho/\delta T$ as a function of temperature showing metal-to-insulator (MI at T_m) and non-trivial insulator-to-metal (IM at T_m*) transitions. (c) MI transition temperature (T_m) and inflection point temperature (T_i) as a function of increasing magnetic field strength. (d) Log_{10} (ρ) versus T^{-1} Arrhenius plots under varying field strengths with the fitting of the insulating regime to obtain gaps. (e) The obtained insulating gaps derived from (d) increase with magnetic-field strength.

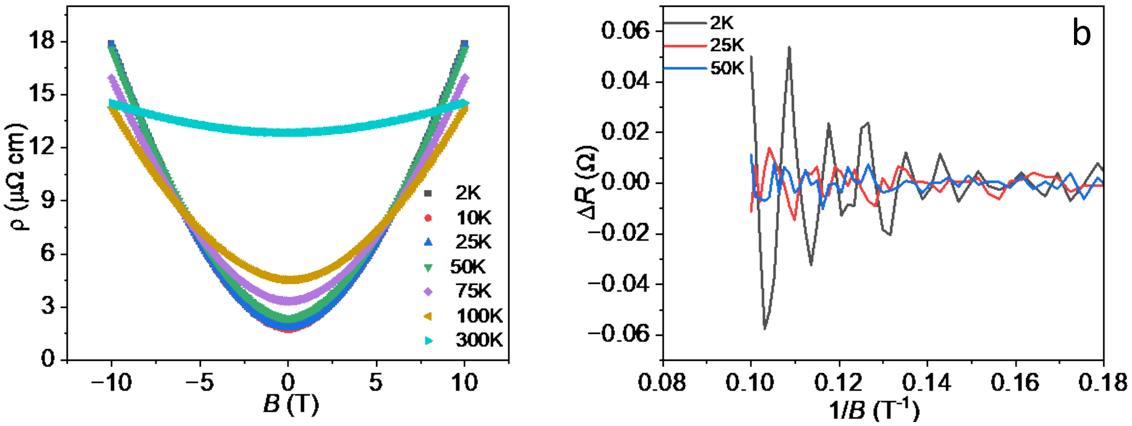

Figure S15. Giant magnetoresistance (GMR) in ~ 270 nm $TaAs_2$ NW device. (a) GMR as a function of temperature. GMR of NW manifest certain robust feature against temperature and decays much slower than the decay observed in bulk $TaAs_2$. (b) Shubnikov-de Hass (SdH)-oscillations as a function of $1/B$ under 2, 25, and 50 K.

Effect of electrical gating on GMR

In compensated semimetals, the magnitude of the MR is related to the degree of carrier compensation. For instance, in thin-WTe_2 flakes, MR was observed to peak at the charge neutrality point.[1] We have studied the effect of gate voltage (V_g) on the MR of our NW devices, employing two different approaches. In the first approach, the device was fabricated on Si/SiO_2 (300 nm SiO_2 with/without 20 nm ALD-HfO_2), and the gate dielectric was a thermal oxide layer on the silicon wafer wherein degenerately doped silicon acted as a back gate (**Figure S16a**). In the second approach, we exploited the *in-situ* grown SiO_2 shell of NWs as the dielectric layer in a top-gate configuration (**Figure S16c**). This approach is simpler and more efficient because the thickness of the SiO_2 shell is 60-80 nm, *i.e.,* ~ 4-5 times thinner than the thermal oxide; hence we expect a stronger field effect. In **Figures S16a, S16c**, we present the effect of V_g on the resistivity of NW devices at 1.8 K under a fixed field of 14 T. In the two device configurations, we observed an increase of MR under a negative to positive V_g scan with an expected relatively faster response in the device with SiO_2-shell as top dielectric (**Figures S16b, S16d**). The increase of MR with positive V_g indicates that the NWs are slightly p-doped; thus, charge compensation occurs under positive V_g. The naturally occurring SiO_2 shell hence exhibits a double benefit: it acts as a removable protective layer for electrical contact and a dielectric layer for gating.

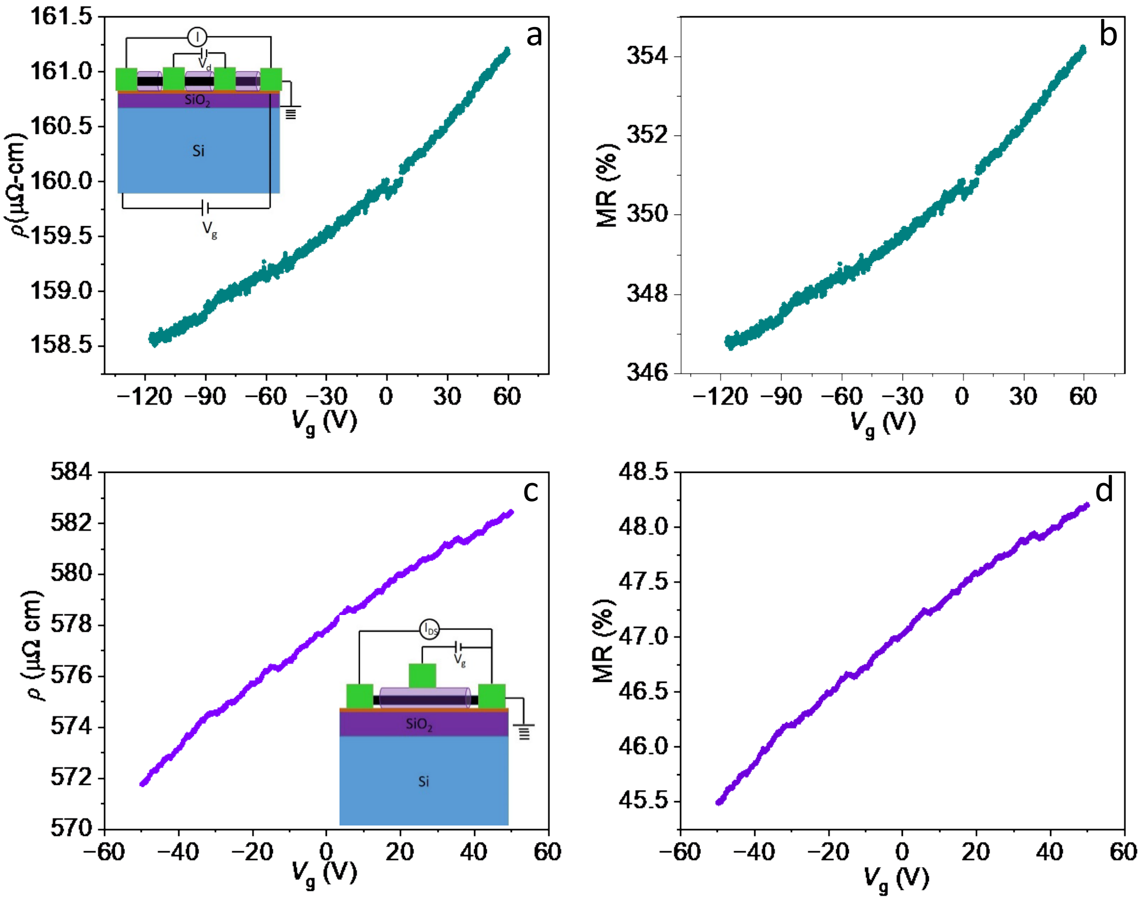

Figure S16. Effect of Gate Voltage (V_g) on the MR of devices. (a) Resistivity as a function of V_g in a $TaAs_2$ NW (d ~ 125 nm) using Si back gate configuration. (b) Corresponding MR change as a function of V_g. (c) Resistivity as a function of V_g in a $TaAs_2$ NW (d ~ 63 nm) using in-situ SiO_2-shell of NW as a top-dielectric. (d) Corresponding MR change as a function of V_g. Gate biasing was applied at a constant B of 14 T at 1.8 K.

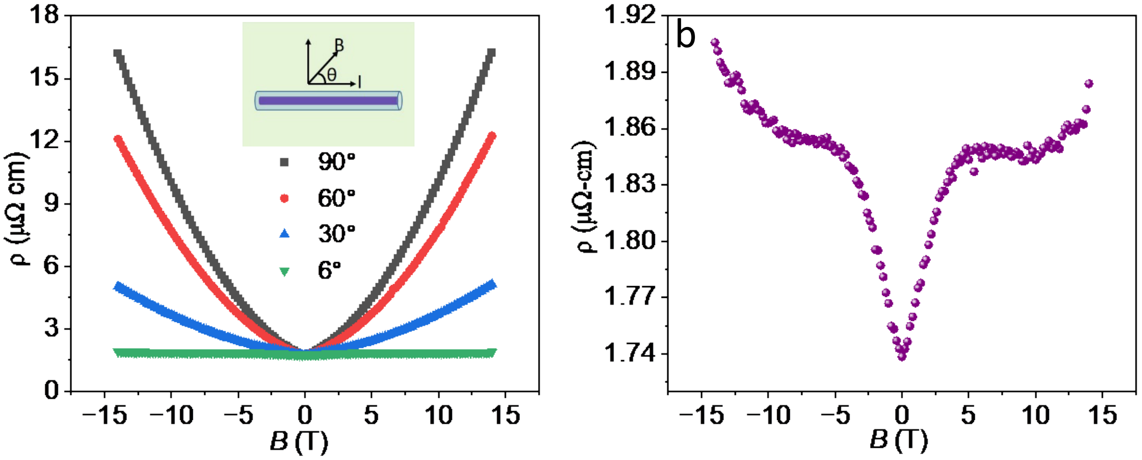

Figure S17. Anisotropic GMR in a 300 nm core $TaAs_2$ NW-device. (a) Magnetoresistance (MR) as a function of the scanned field under the different orientations of the NW-axis with respect to the field direction (*θ*). (b) Resistivity as a function of the scanned field at θ ~ 6° (close to longitudinal field configuration).

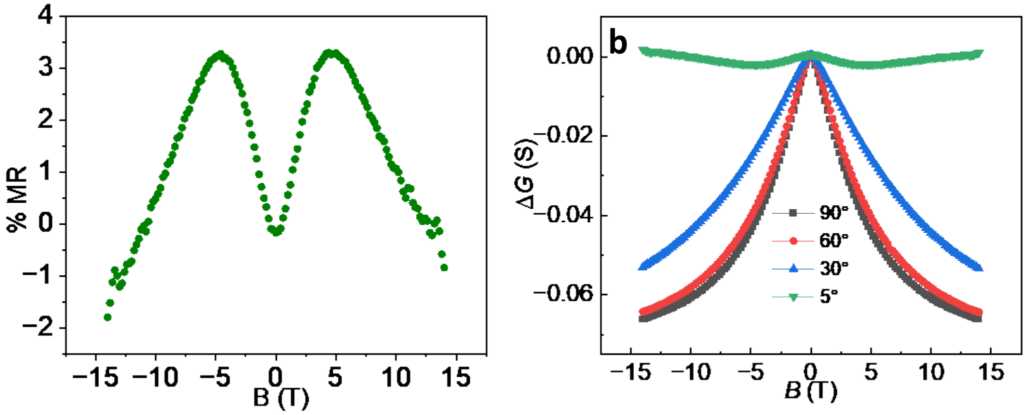

Figure S18. (a) A sharp dip (cusp) in MR near zero-field followed by small-field positive MR turning into negative MR at higher fields. (b) Change in magnetoconductance measured under varying *θ* (angle between the magnetic field and NW-axis or current as shown in **Figure S17a**) from 90 to 5° for a *d* ~ 270 nm NW.

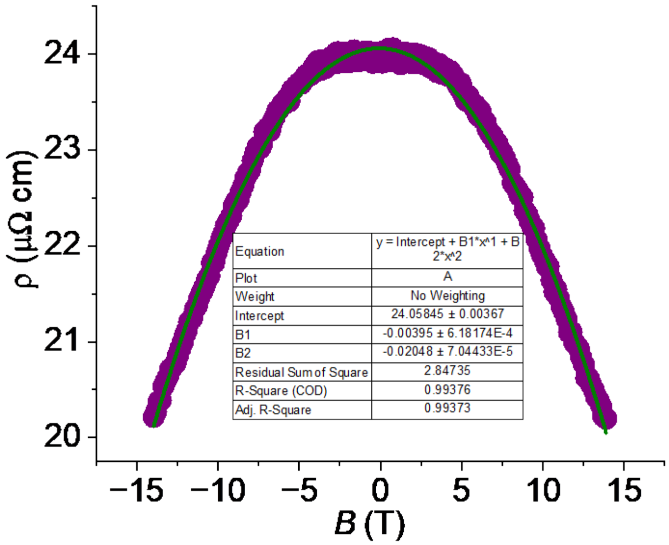

Figure S19. Fitting of field dependent longitudinal negative magnetoresistance curve for a 128 nm $TaAs_2$ NW-device at θ ~ 0° (in a parallel electric and magnetic field configuration).

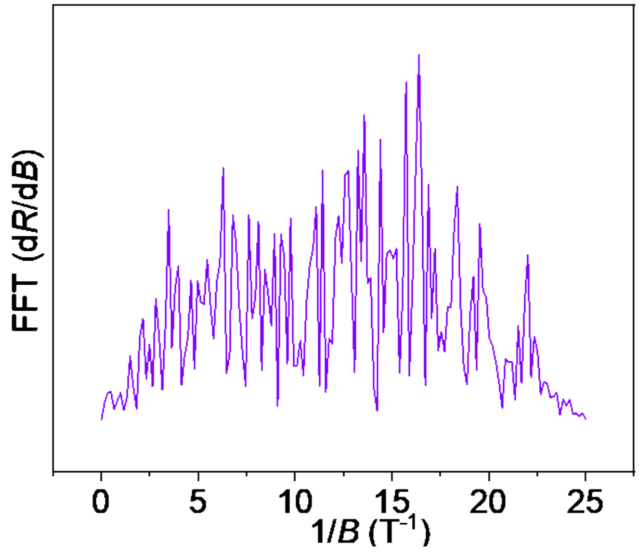

Figure S20. FFT spectrum of MR oscillations in a 128 nm $TaAs_2$ NW showing signatures of universal conductance fluctuation (UCF) and no apparent significant peak.

AB-oscillations from the surface states of a WTI

To gain intuition about the possible origin of a different AB oscillation pattern, we consider a simple model following Ref[8], of two Dirac cones on a two-dimensional surface BZ, away from the **Γ** point, for which the Dirac points are located at two surface momenta $(k_{x1}, k_{y1}), (k_{x2}, k_{y2})$ (**Figures 4i and S21a**). For one of these cones, the low energy Hamiltonian is $\mathrm{H} = \hbar v_x(k_x - \mathrm{k}_{x1})\sigma_x + \hbar v_y(k_y - k_{y1})\sigma_y$, where v_x, v_y represent the Fermi velocities in x and y directions, $\mathbf{k} = (k_x, k_y)$ is the surface momentum, and $\boldsymbol{\sigma}$ are a set of Pauli matrices. In a NW geometry, and under the assumption that the NW is cylindrical with a radius R along the y-axis, k_y remains a good quantum number, however, k_x is now quantized to discrete angular momentum values, which take the form $l_n = n + \frac{1}{2}$ with n an integer. Performing a coordinate transformation, the surface Dirac cones are now discretized to a set of bands that disperse along k_y (**Figure S21b**), and are described by the energies:

$$E_{n,k_1}(k_y, \phi) = \pm\hbar\sqrt{\left[v_x^2\left(\frac{l_n + \phi}{R} - k_{x1}\right)^2 + v_y^2\left(k_y - k_{y1}\right)^2\right]}$$

The NW is threaded with magnetic field along its axis, which is included in the energy spectrum through a shift to the angular momentum: $\phi = \frac{\Phi}{\Phi_0}$ represent the number of magnetic flux quanta through the wire, where $\Phi_0 = \frac{h}{e}$ is a flux quantum and $\Phi = \mathbf{B} \cdot \mathbf{A}$ is the total flux threaded by magnetic field **B** through the wire's cross section **A**.

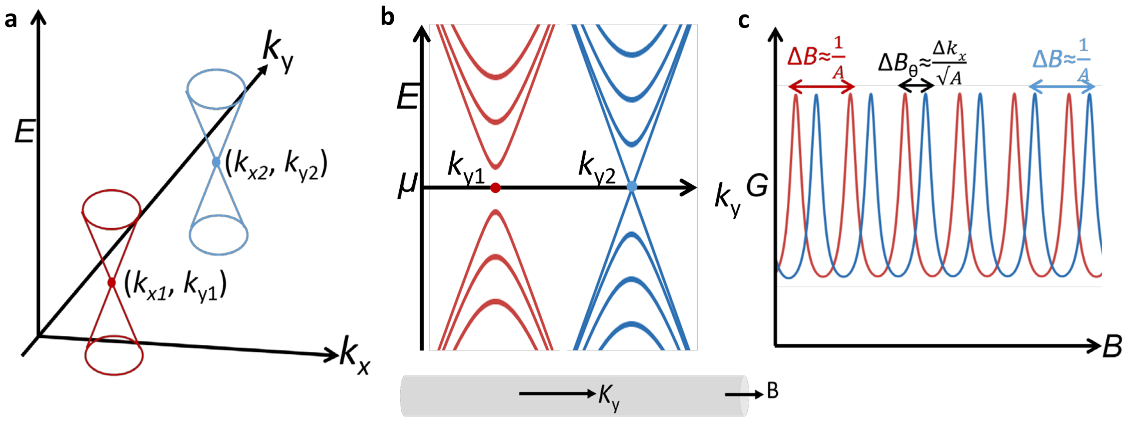

Figure S21. Model of AB oscillations of two surface Dirac cones. (a) Surface spectrum with two Dirac cones located at $(k_{x1}, k_{y1}), (k_{x2}, k_{y2})$. (b) In the NW geometry, the surface Dirac cones become two sets of discrete energy bands, and the magnetic field B shifts them in energy. For general B values the spectrum is gapped, however, for some B values the gap of one or more of the Dirac cones is closed and the bands cross the chemical potential. (c) Each Dirac cone will show oscillations in the conductance G as a function of B, with the same period $\Delta B \sim \frac{1}{A}$ (presented in red and blue). However, between the two periods a phase shift can appear $\Delta B_\theta \sim \frac{\Delta k_x}{\sqrt{A}}$. The total conductance can then get a complex oscillations pattern.

Tuning the magnetic field will change the discrete bands presented (**Figure S21b**), shifting them in energy. Assuming that the chemical potential is located at $E = 0$, the spectrum is generally gapped, however, for some magnetic field values, it is gapless with a linear-dispersing mode crossing the chemical potential. Therefore, the conductance will show oscillations with increasing magnetic fields. (For other chemical potential values, these oscillations will appear due to the change in the of the number of modes[9]. For each Dirac cone, the values in which the spectrum is gapless, namely $E_{n,k_1}(k_y, \phi) = 0$, will appear for momentum $k_y = k_{y1}$ and for magnetic field values

$$\boldsymbol{B_{n,k_1}} = \frac{\Phi_0}{\mathbf{A}}(k_{x1}R - l_n)$$

The period of the oscillations will be the difference between two values of such magnetic fields:

$$\Delta\mathbf{B} = B_{n,k_1} - B_{n+1,k_1} = \frac{\Phi_0}{\mathbf{A}}$$

The period of oscillations for each Dirac cone is therefore inversely proportional to the cross-section, **A**. In the presence of more than one Dirac cone, another oscillation pattern will appear, with the same period (as ΔB is independent of k_{x1}). However, between the two patterns there may be a phase shift:

$$\Delta\mathbf{B}_\theta = \mathrm{B}_{n,k_1} - \mathrm{B}_{n,k_2} = \frac{\Phi_0\sqrt{\pi}}{\sqrt{\mathbf{A}}}\Delta k_x$$

With $\Delta k_x = (k_{x1} - k_{x2})$. Since the total conductance will have contributions from both Dirac cones, it will show a complex AB oscillation pattern, as illustrated in **Figures S21c and 4e**.

We stress that the actual transport measurements will be influenced by many factors not included in this calculation, such as the existence and number of Dirac cones/Fermi arcs in the wire's surfaces' spectra, their location in the BZ, their energy relative to the chemical potential/bulk gap, and the way they connect between the different facets of the NW. However, the model presented here demonstrates how in the presence of a complex surface states spectrum, beyond a single Dirac cone at the **Γ** point, a richer pattern of AB oscillations may appear. Specifically, the double pattern presented here could be interpreted as a pattern with a halved oscillation period, and hence an effective wire cross-section twice larger than the real cross-section, as we see in our results (**Figures 4e and 4f**).

It is interesting to note that the condition for frequency doubling of the AB oscillation pattern depends on the cross-section of the wire. This is because the oscillation period ($\Delta\mathbf{B} = \frac{\Phi_0}{\boldsymbol{A}}$) and a phase shift ($\Delta\mathbf{B}_\theta = \frac{\Phi_0\sqrt{\pi}}{\sqrt{\boldsymbol{A}}}\Delta k_x$) shall scale differently with the nanowire cross-section area *A*. Nanowires having different cross-section areas *A*, are thus expected to have a maximum doubling (manifested as a maximum peak height in the FFT (d*R*/d*B*) as in **Figure 4f**) when the phase shift is half of the oscillation period (or half of it plus an integer multiple of it), namely when $\Delta\mathbf{B}_\theta = \left(n + \frac{1}{2}\right)\Delta B$.This condition would happen for nanowires having a cross-section square root $\sqrt{A} = (\frac{1}{\sqrt{\pi}}\Delta k_x)(n + \frac{1}{2})$, whereas no doubling at all would happen when $\Delta\mathbf{B}_\theta = (n)\Delta B$ and $\sqrt{A} = (\frac{1}{\sqrt{\pi}}\Delta k_x)n$. Any nanowire not having the latter cross-section would show some degree of doubling, though not necessarily a maximum one. This can explain why we observe the frequency doubling in a nanowire having a random cross-section that does not necessarily match the maximum doubling condition.

Although the band structure calculations of bulk $TaAs_2$ and of the TCI phase on the {010} facets have been reported (References 20, 23 and 16 in the manuscript), energy dispersion relations for the WTI phases on the {001}, {201}, and {20$\bar{1}$} facets have not been calculated, as far as we know. Hence the value of Δk_x is not known, and we cannot quantitatively predict what phase shift $\Delta\mathbf{B}_\theta$ should be expected for a $TaAs_2$ NW of cross-section *A* according to our model.

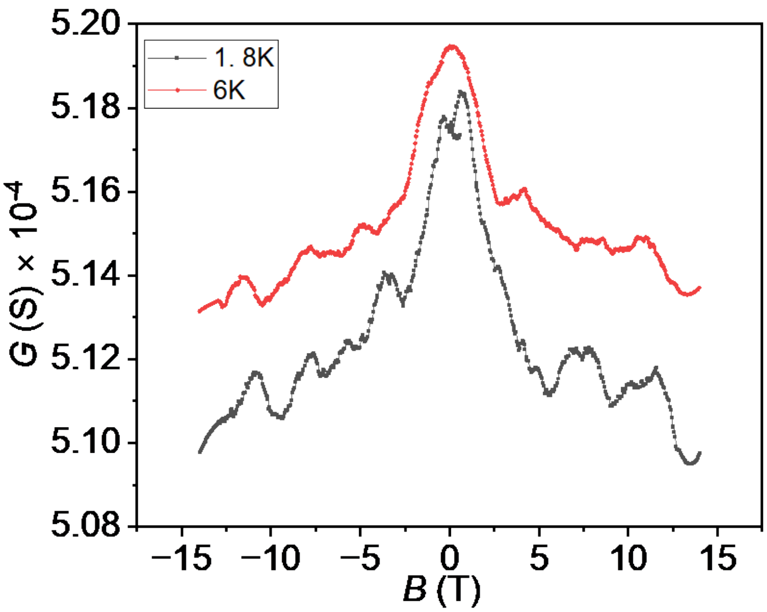

Figure S22. Magnetoconductance at 2 and 6 K as a function of magnetic field for *d*~ 32 nm NW device.

References

[1] C. W. Dunnill, H. K. Edwards, P. D. Brown, D. H. Gregory, *Angew. Chem. Int. Ed.* **2006**, 45, 7060.
[2] E. Sutter, F. Camino, P. Sutter, *Appl. Phys. Lett.* **2009**, 94.
[3] A. Rothman, K. Bukvišová, N. R. Itzhak, I. Kaplan-Ashiri, A. E. Kossoy, X. Sui, L. Novák, T. Šikola, M. Kolíbal, E. Joselevich, *ACS Nano* **2022**, 16, 18757.
[4] Z. Yuan, H. Lu, Y. Liu, J. Wang, S. Jia, *Phys. Rev. B* **2016**, 93, 184405.
[5] Y.-Y. Wang, Q.-H. Yu, P.-J. Guo, K. Liu, T.-L. Xia, *Phys. Rev. B* **2016**, 94, 041103.
[6] A. S. Wadge, G. Grabecki, C. Autieri, B. J. Kowalski, P. Iwanowski, G. Cuono, M. F. Islam, C. M. Canali, K. Dybko, A. Hruban, A. Łusakowski, T. Wojciechowski, R. Diduszko, A. Lynnyk, N. Olszowska, M. Rosmus, J. Kołodziej, A. Wiśniewski, *J. Phys.: Condens. Matter* **2022**, 34, 125601.
[7] Y. Luo, R. D. McDonald, P. F. S. Rosa, B. Scott, N. Wakeham, N. J. Ghimire, E. D. Bauer, J. D. Thompson, F. Ronning, *Sci. Rep.* **2016**, 6, 27294.

[8] d. J. F. Majlin Skiff R, Queiroz R, Mathimalar S, Beidenkopf H, Ilan R., *SciPost Physics Core.* **2023**, 6, 011.
[9] J. H. Bardarson, J. E. Moore, *Rep. Prog. Phys.* **2013**, 76, 056501.
